# Validation is not enough: Longitudinal evidence of post-deployment fragility in clinical AI systems

**DOI:** 10.1371/journal.pdig.0001534

**Published:** 2026-07-27

**Authors:** Georgy Kopanitsa

**Affiliations:** 1 Federal State Budgetary Institution, V.A. Almazov National Medical Research Centre of the Ministry of Health of the Russian Federation, Saint-Petersburg, Russia; 2 ITMO University, Saint-Petersburg, Russia; ETH Zurich, SWITZERLAND

## Abstract

Pre-deployment validation is commonly used to establish the safety and effectiveness of clinical artificial intelligence systems, but acceptable validation performance does not guarantee stable behavior after deployment into routine clinical workflows. We conducted a longitudinal retrospective observational study of four clinically deployed AI systems operating across distinct clinical domains and workflows within a large healthcare organization. Using routinely collected clinical data, outcome labels, and operational telemetry, we compared validation-era performance with post-deployment behavior over extended observation periods. Analyses focused on temporal patterns of discrimination, calibration, data availability, latency, and workflow-related signals, with particular attention to label-dependent and label-independent monitoring. Across all systems, validation-era performance did not persist as a stable operational property after deployment. Calibration drift emerged consistently and often preceded detectable changes in discrimination. Workflow-associated changes in data availability and timing were more strongly and consistently associated with degradation than population-level indicators. Label-independent operational signals, including input missingness and data latency, provided early indication of emerging fragility, whereas outcome-based monitoring was delayed by label latency and documentation processes. These findings suggest that post-deployment fragility can be a structural property of clinical AI systems embedded in evolving workflows. Effective governance therefore requires lifecycle-oriented monitoring strategies that combine calibration reassessment with operational telemetry throughout deployment.

## Author summary

Artificial intelligence systems are increasingly used in clinical care, but most are assessed before deployment using historical data. This type of validation can show that a system performed well in the past, but it does not prove that the system will remain reliable after it becomes part of everyday clinical work. In this study, we examined four clinical AI systems after deployment and compared their real-world behavior with their validation performance. We found that performance changed over time even though the models themselves were not intentionally modified. The most consistent early problem was not always a drop in the model’s ability to rank patients by risk, but a loss of calibration, meaning that the predicted risk no longer matched the observed risk. We also found that changes in data availability and timing, such as missing inputs or delayed data reaching the AI system, were useful early warning signals. These findings suggest that clinical AI systems need ongoing monitoring after deployment, especially using operational signals that are available before outcome labels are complete.

## 1. Introduction

Clinical artificial intelligence systems are most commonly justified for clinical use through a static validation paradigm, in which a model is developed and evaluated on retrospective data and its performance summarized using a small set of global metrics, such as discrimination and calibration. These validation results are then treated – implicitly or explicitly – as evidence that the model will remain reliable after deployment. This paradigm is deeply embedded across the clinical AI lifecycle: it shapes how studies are designed and reported, how regulatory and procurement decisions are made, and how risks are communicated to clinical stakeholders.

In practice, validation usually takes one of three forms: (1) internal validation using a held-out test set drawn from the same data-generating process as the training data [1]; (2) temporal validation using a later time window within the same health system [2]; or (3) external validation in a different institution or setting [3]. Each of these approaches is methodologically appropriate for estimating performance under conditions similar to the evaluation data. However, clinical deployment rarely preserves these conditions. Once embedded into routine care, AI systems become tightly coupled to evolving clinical workflows, documentation practices, information system configurations, and care pathways [4,5]. Clinical practice patterns learned from EHR data can vary substantially across years [6]. These changes may occur gradually or abruptly, often without any explicit modification to the model itself, creating the potential for silent performance degradation, in which predictions continue to be generated despite a loss of alignment between model inputs, outputs, and their clinical meaning.

The limitation of static validation has been widely acknowledged in principle. In the machine learning literature, this problem is formalized under the umbrella of dataset shift or concept drift, encompassing changes in the joint distribution of inputs, outcomes, and their relationships [7]. In healthcare, several authors have emphasized that such shifts are not exceptional events but a structural property of clinical data. Finlayson et al. argue that dataset shift in medicine is “the rule rather than the exception,” driven by changes in care delivery, clinical incentives, and documentation rather than by random variation alone [8]. This observation reframes degradation from a purely technical challenge into a problem of governance and safety: if change is inevitable, then one-time validation cannot provide durable assurance of clinical reliability.

Empirical evidence increasingly supports this concern. Longitudinal analyses have shown that predictive models may lose calibration and discrimination over time within the same institution, even when patient demographics and nominal case severity remain relatively stable [9]. Minne et al. showed that the performance of a customized SAPS-II ICU mortality model changed over time, illustrating how temporal changes in clinical case mix and care processes can affect the reliability of severity-adjustment models [10]. Care-process shifts – especially changes in test-ordering and documentation practices – can alter both feature availability and the meaning of missingness (“informative missingness”), which can affect deterioration-prediction performance even when underlying patient mix is relatively stable [11,12]. These studies suggest that performance decay may be driven as much by changes in clinical processes as by changes in patient populations, yet such mechanisms are rarely measured explicitly.

High-profile failures of generalization further illustrate the fragility of relying on validation metrics alone [13]. In medical imaging, Zech et al. showed that a pneumonia detection model learned site-specific and non-clinical confounders, resulting in large performance differences across hospitals despite strong internal validation [14]. Similarly, external evaluations of widely deployed proprietary systems, such as the Epic Sepsis Model, have reported poor discrimination and calibration in routine clinical use despite broad adoption [15,16]. While these examples are often framed as transportability failures, they also foreshadow what may occur longitudinally within a single institution as scanners, protocols, coding practices, and workflows evolve over time.

An important but comparatively underexplored contributor to degradation is workflow-induced drift [17]. Clinical data are not passively observed, they are produced through sociotechnical processes involving clinicians, order sets, templates, interfaces, and organizational policies. Changes in these processes can alter not only feature distributions but also their semantic interpretation. Subbaswamy and Saria emphasize that predictive systems that do not explicitly account for causal structure are brittle to policy and workflow changes, even when statistical properties appear similar [18]. Complementary work on alert fatigue and automation bias shows that clinician interaction with decision support systems can recursively influence both inputs and outcomes, creating feedback loops that invalidate original performance assumptions [19,20].

Regulatory and reporting guidance increasingly acknowledges the need for lifecycle-based evaluation of AI systems [21–23]. Frameworks such as TRIPOD-AI and CONSORT-AI have substantially improved transparency in model development and validation, and regulatory bodies emphasize ongoing monitoring for AI/ML-enabled medical devices [24]. However, these frameworks primarily focus on evidence generation at the point of evaluation and provide limited guidance on how performance degradation should be detected and managed during routine clinical operation, particularly when outcome labels are delayed, incomplete, or influenced by the model’s own use [25–27].

Taken together, existing literature establishes a clear consensus that model degradation is expected, common, and potentially harmful. What remains insufficiently understood is how degradation manifests during real-world deployment, which drift mechanisms are most influential under routine operating conditions, and which observable signals provide early warning of safety-relevant failure. Addressing this gap requires moving beyond snapshot validation toward longitudinal, post-deployment evaluation that explicitly accounts for temporal, population, and workflow-related change.

This paper is positioned as a methodological and empirical governance contribution, rather than as a benchmarking study of models or tasks. The central objective is to characterize the structure of post-deployment fragility – how divergence from validation emerges over time, which mechanisms plausibly drive it in routine operation, and which monitoring signals are realistically observable under governance constraints such as delayed or biased outcome labels. Accordingly, the analyses emphasize longitudinal patterns, failure modes, and observability, and treat performance metrics as operational evidence to support governance-relevant claims rather than as the primary endpoint.

### 1.1. Study objectives and analytical focus

The objective of this study is to empirically characterize how clinically deployed AI systems diverge from their validation-era behavior during routine operation, and to identify which forms of change are most relevant for *governance and safety assurance* after deployment. Rather than evaluating which models or tasks perform best, the study focuses on understanding *how and why* performance degradation emerges over time, which mechanisms plausibly drive it under real-world operating conditions, and which signals are realistically observable within existing clinical information systems.

Specifically, the study is designed to address the gap between pre-deployment evaluation and post-deployment reliability by comparing model performance during validation with performance observed under real-world operating conditions over time. In doing so, it seeks to complement existing work on dataset shift and transportability with empirical evidence derived from routine clinical use, where changes in workflows, data availability, and system configuration are common and often undocumented.

### 1.2. Primary analytical objective

The primary analytical objective is to characterize the *magnitude, timing, and structure* of divergence between validation-era performance and post-deployment behavior of clinical AI systems. Emphasis is placed on how this divergence evolves longitudinally during routine operation and on which performance properties – particularly calibration – exhibit early and safety-relevant instability under real-world conditions.

### 1.3. Secondary analytical objectives

To support interpretation and practical relevance, three secondary objectives are pursued:

1. To characterize distinct sources of performance change by decomposing observed degradation into temporal, population-related, and workflow-associated components. This analysis responds to calls for more granular understanding of dataset shift mechanisms in healthcare settings.
2. To examine the relationship between operational data integrity indicators and model performance, including measures of input missingness, data latency, and protocol-driven feature availability. This objective builds on prior informatics research highlighting the impact of data quality and workflow design on clinical decision support effectiveness [28–30].
3. To explore the feasibility of label-independent monitoring signals as early indicators of performance degradation, acknowledging that outcome labels in clinical practice are often delayed, incomplete, or influenced by system use itself.

### 1.4. Analytical hypotheses

- **Hypothesis 1.** Clinical AI systems that meet predefined validation criteria will exhibit *operationally and governance-relevant divergence* from validation-era behavior after deployment, even when model parameters, feature definitions, and intended use remain unchanged.
- **Hypothesis 2.** Workflow-associated and operational changes in data generation, availability, and timing will show stronger associations with post-deployment performance divergence than population-level indicators alone, highlighting workflow-induced drift as a primary governance concern.
- **Hypothesis 3.** Label-independent operational indicators observable at inference time – such as input missingness and data latency – will precede or co-evolve with safety-relevant performance degradation, supporting their use as early-warning signals under real-world governance constraints.

## 2. Methods

### 2.1. Ethics statement

This retrospective study was reviewed and approved by the local Almazov Center Ethics Committee. The requirement for informed consent was waived because the study used retrospective data and posed minimal risk to participants. Access to clinical and operational data was governed by institutional data-use procedures. Analyses were conducted on restricted-access data, and reported outputs were limited to de-identified aggregate results. All data were handled in accordance with institutional policies and applicable privacy and data-protection regulations.

### 2.2. Study design

We conducted a retrospective, longitudinal observational study of clinically deployed AI systems to evaluate post-deployment behavior under routine operating conditions. The study compared model performance observed during pre-deployment validation with performance measured after deployment and examined how performance evolved over time in relation to temporal, population-related, and workflow-associated factors.

An observational design was chosen to reflect real-world clinical practice. No changes were made to model parameters, feature definitions, decision thresholds, or intended use during the observation period. All systems were evaluated as implemented within existing clinical workflows.

#### 2.2.1. Study phases and unit of analysis.

For each AI system, the evaluation period was divided into three analytically distinct phases.

- **The validation phase** corresponded to the dataset and time window used for internal or temporal validation before deployment.
- **The early deployment phase** was defined in the primary analysis as the first 6 months after clinical deployment, corresponding to the initial post-integration period during which workflows and data pipelines were expected to stabilize.
- **The mature deployment** phase was defined as the period after the first 6 months of deployment, corresponding to sustained routine operation.

Sensitivity analyses repeated the phase-level comparisons using alternative early deployment definitions of 3 and 9 months, with the 6-month definition used as the primary analysis.

These analyses were used to evaluate the robustness of the validation–deployment comparison to shorter and longer assumptions about the initial post-integration period.

The primary unit of analysis was the individual inference event, defined as a single execution of the AI system producing a prediction for a specific patient encounter. Longitudinal analyses were performed using temporally aggregated system-level estimates.

For deployment-phase sensitivity analyses, performance differences were calculated as the early deployment estimate minus the validation-phase estimate for each system and phase definition. System-specific differences were then summarized as mean differences across systems with 95% confidence intervals. Negative values indicate lower post-deployment performance compared with validation.

#### 2.2.2. Outcomes and scope.

The study focused on model performance and operational behavior, rather than downstream patient outcomes. This choice reflects the study’s aim to evaluate reliability and safety signals that are observable during routine operation, independent of delayed or incomplete clinical outcome labels. Patient management decisions were not altered for the purpose of this study, and no prospective clinical interventions were introduced.

### 2.3. Clinical AI systems and use contexts

Four clinically deployed AI systems (Systems A–D) were included. Systems were anonymized to preserve institutional confidentiality and to avoid conflating governance-relevant findings with vendor-specific implementations.

Eligible systems were those deployed in routine clinical practice with documented pre-deployment validation, reliance on standard clinical data sources (EHR, laboratory, or imaging systems), and sufficient post-deployment operational data to support longitudinal analysis. Systems undergoing retraining, threshold modification, or changes in intended use during the observation period were excluded.

The included systems spanned heterogeneous clinical domains, data modalities, and workflow contexts, including high-frequency EHR-based predictive models and lower-frequency diagnostic and imaging-based applications. Anonymization reflects the study’s focus on post-deployment behavior as a sociotechnical phenomenon rather than comparative benchmarking of specific commercial products.

#### 2.3.1. Inclusion criteria for AI systems.

AI systems were eligible for inclusion if they met the following criteria:

1. The system was deployed in routine clinical practice and used to generate predictions or risk scores for real patient encounters.
2. The model had undergone formal pre-deployment validation, with documented performance metrics available from internal or temporal validation.
3. The system relied on data sources available through standard clinical information systems (e.g., EHR, laboratory information systems, imaging archives).
4. Sufficient post-deployment operational data were available to support longitudinal analysis of performance and data integrity indicators.

Systems that were purely experimental, used only in silent mode without generating outputs, or subject to frequent retraining or threshold changes during the study period were excluded to avoid conflating degradation with intentional model modification.

### 2.4. Data sources and observability

Analyses used routinely collected clinical, outcome, and operational data generated during validation and routine deployment. Clinical inputs were obtained from institutional EHRs, laboratory systems, and, where applicable, imaging-derived repositories, and were extracted as available at the time of inference to preserve real-world temporal constraints.

Outcome labels were derived from routine clinical documentation and were consistent with those used during validation. Because outcome availability varied by task and was subject to workflow-related delays, these constraints were explicitly considered in interpreting label-dependent monitoring.

Operational metadata were treated as primary data sources and included inference timestamps, input availability and missingness, data latency, and system interaction logs where available. These signals are observable during routine operation and do not depend on outcome adjudication.

All data sources were linked at the inference level and temporally aligned relative to prediction time. Minimal preprocessing was applied, and feature handling remained consistent with original model implementation.

### 2.5. Drift taxonomy and analytical framework

Post-deployment performance change was analyzed using a pragmatic drift taxonomy distinguishing three non-mutually exclusive categories:

- Temporal drift, capturing time-dependent performance changes not attributable to explicit population or workflow shifts;
- Population drift, reflecting changes in patient demographics, case mix, or outcome prevalence;
- Workflow-induced drift, reflecting changes in data availability, timing, or semantic meaning arising from workflow evolution, documentation practices, or system configuration.

Population drift was operationalized using routinely available patient-level variables. Workflow-induced drift was assessed using operational indicators such as input missingness, data latency, and feature availability. These categories were used as an analytical framework rather than as mutually exclusive causal labels.

### 2.6. Performance and operational metrics

Predictive performance was evaluated using the same primary metrics applied during validation to ensure comparability, including discrimination (AUROC and, where appropriate, AUPRC), calibration (slope and intercept), and threshold-dependent measures defined at deployment and kept constant throughout the study. Metrics were calculated within predefined temporal intervals, with confidence intervals estimated via bootstrap resampling.

Operational indicators were quantified to assess changes in data integrity and workflow execution, including input missingness, data latency, feature staleness, inference volume, and system interaction measures where available. These indicators were summarized over the same temporal intervals as performance metrics to enable direct comparison.

### 2.7. Statistical analysis

Statistical analyses were designed to quantify longitudinal divergence between validation-era and post-deployment model behavior and to examine whether this divergence was associated with temporal, population-related, or workflow-associated indicators [31,32].

All analyses were performed separately for each AI system and, where specified, in pooled models including all systems.

The individual inference event was the base unit of analysis. For longitudinal analyses, inference events were aggregated by system and post-deployment time interval. Monthly aggregation was used for the main longitudinal trajectories. Phase-level analyses compared validation, early deployment, and mature deployment periods.

For each system and time interval, we calculated AUROC, AUPRC where applicable, calibration slope, calibration intercept, input missingness, data latency, feature availability, inference volume, and available system-interaction measures. Confidence intervals for performance metrics were estimated using bootstrap resampling within each system and time interval.

Longitudinal performance trajectories were summarized using monthly estimates and rolling-window analyses. Sensitivity analyses repeated the longitudinal analyses using alternative temporal aggregation windows to assess whether the observed direction and timing of performance degradation depended on the selected window size.

Deployment-phase comparisons used the primary validation, early deployment, and mature deployment definitions specified in Section 2.1. Sensitivity analyses repeated the phase-level comparisons using alternative early deployment definitions of 3 and 9 months, with the 6-month definition used as the primary analysis.

Regression analyses were performed at the system-time-interval level, where each observation represented one AI system within one post-deployment temporal interval. The primary dependent variable for drift-association analyses was calibration slope, selected a priori because calibration reflects the reliability of absolute risk estimates used in threshold-based clinical decision support. Secondary dependent variables were calibration intercept, AUROC, and AUPRC where applicable.

Population-related indicators included demographic composition, available case-mix variables, and outcome prevalence. Workflow-associated indicators included input missingness, data latency, feature availability, inference volume, and available interaction measures. Continuous covariates were standardized before regression modelling.

The primary pooled model used a mixed-effects linear regression with a system-level random intercept. The general model form was:


$$\begin{array}{c} {Performance}_{s,t}\;=\;\beta_{\text{0}}\;+\;\beta_{\text{1}}{Time}_{s,t}\;+\;\beta_{\text{2}}\;{PopulationIndicators}_{s,t}\;+\;\beta_{\text{3}}{WorkflowIndicators}_{s,t}\;+\;u_{s}\;+\;\varepsilon_{s,t,} \end{array}$$

where *s* denotes the AI system, *t* denotes the post-deployment time interval, *u*s is the system-level random intercept, and εs,t is the residual error.

To compare population-related and workflow-associated indicators, three model blocks were estimated: a population-only block, a workflow-only block, and a combined population-plus-workflow block. The relative contribution of each block was summarized using standardized regression coefficients, 95% confidence intervals, changes in explained variance, and consistency of effect direction across systems and robustness specifications. These analyses were interpreted as associations between covariate blocks and performance degradation, not as causal decomposition.

Lagged analyses evaluated whether operational telemetry indicators in preceding time intervals were associated with subsequent performance metrics. For each system, one-interval and two-interval lagged versions of missingness, latency, and feature availability were created. These lagged telemetry variables were then related to subsequent calibration slope, calibration intercept, AUROC, and AUPRC using the same system-time-interval structure and mixed-effects framework as the primary regression analyses. Lagged analyses were used to assess temporal ordering between telemetry change and later performance degradation.

For system-specific drift analyses, calibration slope was used as the primary dependent variable because calibration degradation was the earliest and most consistent post-deployment failure pattern. Associations between candidate drift indicators and calibration slope were estimated using standardized regression coefficients. Continuous drift indicators were standardized before model fitting. Coefficients are reported as standardized β estimates with 95% confidence intervals. Negative coefficients indicate an association between higher drift-indicator values and lower calibration slope.

System-specific models were used to generate the standardized coefficients reported in Table 1, while pooled mixed-effects models and alternative model specifications were used for the robustness analyses summarized in Tables 2 and Table 3.

**Table 1:** Associations between candidate drift indicators and post-deployment calibration degradation.

Drift indicator	System A βstd (95% CI)	System B βstd (95% CI)	System C βstd (95% CI)	System D βstd (95% CI)
**Population demographics shift**	-0.05 (-0.12 to 0.03)	-0.04 (-0.11 to 0.04)	-0.03 (-0.10 to 0.05)	-0.06 (-0.14 to 0.02)
**Outcome prevalence shift**	-0.18 (-0.28 to -0.08)	-0.16 (-0.26 to -0.06)	-0.07 (-0.16 to 0.02)	-0.08 (-0.18 to 0.01)
**Input missingness**	-0.42 (-0.56 to -0.29)	-0.38 (-0.52 to -0.25)	-0.24 (-0.37 to -0.11)	-0.21 (-0.34 to -0.08)
**Data latency**	-0.35 (-0.49 to -0.21)	-0.27 (-0.40 to -0.13)	-0.08 (-0.18 to 0.02)	-0.23 (-0.36 to -0.10)
**Feature availability change**	-0.22 (-0.35 to -0.09)	-0.34 (-0.48 to -0.20)	-0.19 (-0.31 to -0.07)	-0.37 (-0.51 to -0.23)
**Alert acknowledgement/ override**	-0.07 (-0.16 to 0.02)	-0.06 (-0.15 to 0.03)	NA	NA

**Table 2:** Leave-one-system-out robustness of telemetry vs population drift associations.

Excluded system	β (missingness)	β (latency)	β (population drift index)	ΔR² telemetry block	ΔR² population block	Telemetry > population	Temporal ordering preserved*
Exclude A	−0.082 [−0.102, −0.061]	−0.041 [−0.056, −0.027]	−0.012 [−0.028, 0.004]	0.21	0.06	Yes	Yes
Exclude B	−0.094 [−0.115, −0.073]	−0.048 [−0.063, −0.033]	−0.008 [−0.024, 0.007]	0.24	0.05	Yes	Yes
Exclude C	−0.101 [−0.123, −0.079]	−0.053 [−0.069, −0.038]	−0.015 [−0.031, 0.002]	0.27	0.07	Yes	Yes
Exclude D	−0.108 [−0.131, −0.086]	−0.057 [−0.074, −0.041]	−0.010 [−0.026, 0.006]	0.28	0.06	Yes	Yes

**Table 3:** Robustness of telemetry–performance associations under alternative model specifications.

Signal category	βstd range across specifications	Specifications preserving expected direction	Median ΔR²	Robustness interpretation
Input missingness	-0.46 to -0.31	12/12	0.18	Consistent negative association
Data latency	-0.39 to -0.24	12/12	0.13	Consistent negative association
Feature availability change	-0.35 to -0.18	10/12	0.09	Moderately consistent negative association
Population demographics	-0.09 to 0.04	6/12	0.03	Inconsistent association

Robustness analyses included leave-one-system-out models and alternative covariate specifications. For alternative regression specifications, robustness was summarized using the range of standardized regression coefficients across specifications, the proportion of specifications preserving the expected direction of association, and the median incremental explained variance associated with each signal category. Sensitivity analyses included alternative deployment-phase definitions, alternative temporal aggregation windows, and alternative regression specifications.

Results were summarized using effect estimates, 95% confidence intervals, explained-variance changes, and consistency across systems and robustness specifications.

Analyses were performed in Python 3.11.7. Data processing and temporal aggregation were performed using pandas 2.1.4 and NumPy 1.26.4. Performance metrics, including AUROC and AUPRC, were calculated using scikit-learn 1.3.2. Calibration slope and calibration intercept were estimated using statsmodels 0.14.1. Bootstrap confidence intervals, regression analyses, lagged analyses, sensitivity analyses, and leave-one-system-out robustness analyses were implemented using NumPy 1.26.4, pandas 2.1.4, scikit-learn 1.3.2, statsmodels 0.14.1, and SciPy 1.11.4. Figures were generated using matplotlib 3.8.2. All scripts were maintained under version control.

## 3. Results

### 3.1. Empirical cohort and deployment characteristics

The empirical evaluation included four clinically deployed AI systems operating across heterogeneous clinical domains, data modalities, and workflow contexts.

In the primary analysis, early deployment refers to the first 6 months after deployment, and mature deployment refers to the subsequent observation period.

Additional system descriptors are reported to support interpretation of transferability across clinical contexts while preserving anonymization of local implementations, vendors, and institution-specific workflow details.

Systems A and B were high-frequency EHR-based predictive models deployed in acute care settings. System C was a laboratory and structured-EHR-based diagnostic decision-support system used in specialty outpatient care. System D was an imaging-derived outcome-risk model embedded in the radiology workflow. Observation periods ranged from 18 to 24 months, and inference volumes ranged from 68,000–1,240,000 events.

Outcome-label availability differed substantially across systems. Acute-care outcomes were typically available within hours to days, whereas diagnostic and imaging outcomes were subject to delays of weeks. Across all systems, model architecture, feature definitions, decision thresholds, and intended use remained unchanged during the observation period. Therefore, observed changes in model behavior reflected post-deployment operating conditions rather than intentional model modification.

### 3.2. Validation versus post-deployment performance

Validation-era performance met predefined acceptance criteria for all systems and served as the reference for post-deployment comparison. In the primary phase-level analysis, early deployment was defined as the first 6 months after clinical deployment, and mature deployment as the period after the first 6 months (Table 4).

**Table 4:** Predictive performance across validation, early deployment, and mature deployment phases.

System ID	Phase	Temporal definition	AUROC	AUPRC	Calibration slope	Calibration intercept
System A	Validation	Pre-deployment validation dataset/time window	0.82 (0.81–0.83)	0.38 (0.36–0.40)	1.01 (0.97–1.05)	0.02 (-0.01 – 0.05)
	Early deployment	Months 0–5 after deployment	0.81 (0.79–0.82)	0.36 (0.34–0.38)	0.88 (0.82–0.94)	-0.18 (-0.24 – -0.12)
	Mature deployment	Month 6 onward	0.78 (0.76–0.80)	0.32 (0.30–0.34)	0.71 (0.65–0.77)	-0.36 (-0.42 – -0.30)
System B	Validation	Pre-deployment validation dataset/time window	0.80 (0.78–0.82)	0.29 (0.27–0.31)	0.99 (0.93–1.05)	0.01 (-0.04 –0.06)
	Early deployment	Months 0–5 after deployment	0.79 (0.77–0.81)	0.27 (0.25–0.29)	0.86 (0.79–0.93)	-0.21 (-0.28 – -0.14)
	Mature deployment	Month 6 onward	0.75 (0.73–0.77)	0.24 (0.22–0.26)	0.69 (0.62–0.76)	-0.41 (-0.48 – -0.34)
System C	Validation	Pre-deployment validation dataset/time window	0.84 (0.82–0.86)	0.42 (0.39–0.45)	1.02 (0.96–1.08)	-0.01 (-0.05 –0.03)
	Early deployment	Months 0–5 after deployment	0.83 (0.80–0.85)	0.41 (0.38–0.44)	0.93 (0.86–1.00)	-0.09 (-0.15 – -0.03)
	Mature deployment	Month 6 onward	0.82 (0.79–0.84)	0.40 (0.37–0.43)	0.85 (0.78–0.92)	-0.17 (-0.23 – -0.11)
System D	Validation	Pre-deployment validation dataset/time window	0.86 (0.84–0.88)	0.46 (0.43–0.49)	1.03 (0.97–1.09)	0.00 (-0.04 –0.04)
	Early deployment	Months 0–5 after deployment	0.83 (0.81–0.85)	0.43 (0.40–0.46)	0.95 (0.88–1.02)	-0.08 (-0.14 – -0.02)
	Mature deployment	Month 6 onward	0.79 (0.77–0.81)	0.39 (0.36–0.42)	0.89 (0.82–0.96)	-0.19 (-0.25 – -0.13)

Across systems, post-deployment performance diverged from validation-era estimates despite unchanged model parameters, feature definitions, decision thresholds, and intended use. Calibration changed earlier and more consistently than discrimination. In Systems A and B, AUROC remained close to validation levels during early deployment, whereas calibration slopes declined and calibration intercepts shifted. In mature deployment, calibration degradation was more pronounced across Systems A–C, while System D showed a larger decline in discrimination.

These findings indicate that discrimination metrics alone would have delayed detection of post-deployment degradation, particularly in Systems A and B, where calibration drift emerged before substantial AUROC decline.

### 3.3. Quantitative associations between drift indicators and calibration degradation

Associations between candidate drift indicators and calibration degradation are summarized in Table 1. Workflow-associated indicators showed larger and more consistent negative associations with calibration slope than population-related indicators. Input missingness and data latency were most strongly associated with declining calibration slope in Systems A and B. Feature availability change showed the strongest association in Systems B and D. Population demographics showed weak and inconsistent associations across all systems, while outcome prevalence showed modest associations in Systems A and B.

βstd denotes the standardized regression coefficient from system-specific regression models with calibration slope as the dependent variable. Continuous drift indicators were standardized before model fitting. Negative coefficients indicate that higher values of the drift indicator were associated with lower calibration slope. Values are reported with 95% confidence intervals. NA indicates that the corresponding telemetry signal was not available or not applicable for that system.

Overall, workflow-associated indicators showed stronger and more consistent associations with post-deployment calibration degradation than population-related indicators.

### 3.4. Longitudinal performance and telemetry trajectories

**Fig 1:**
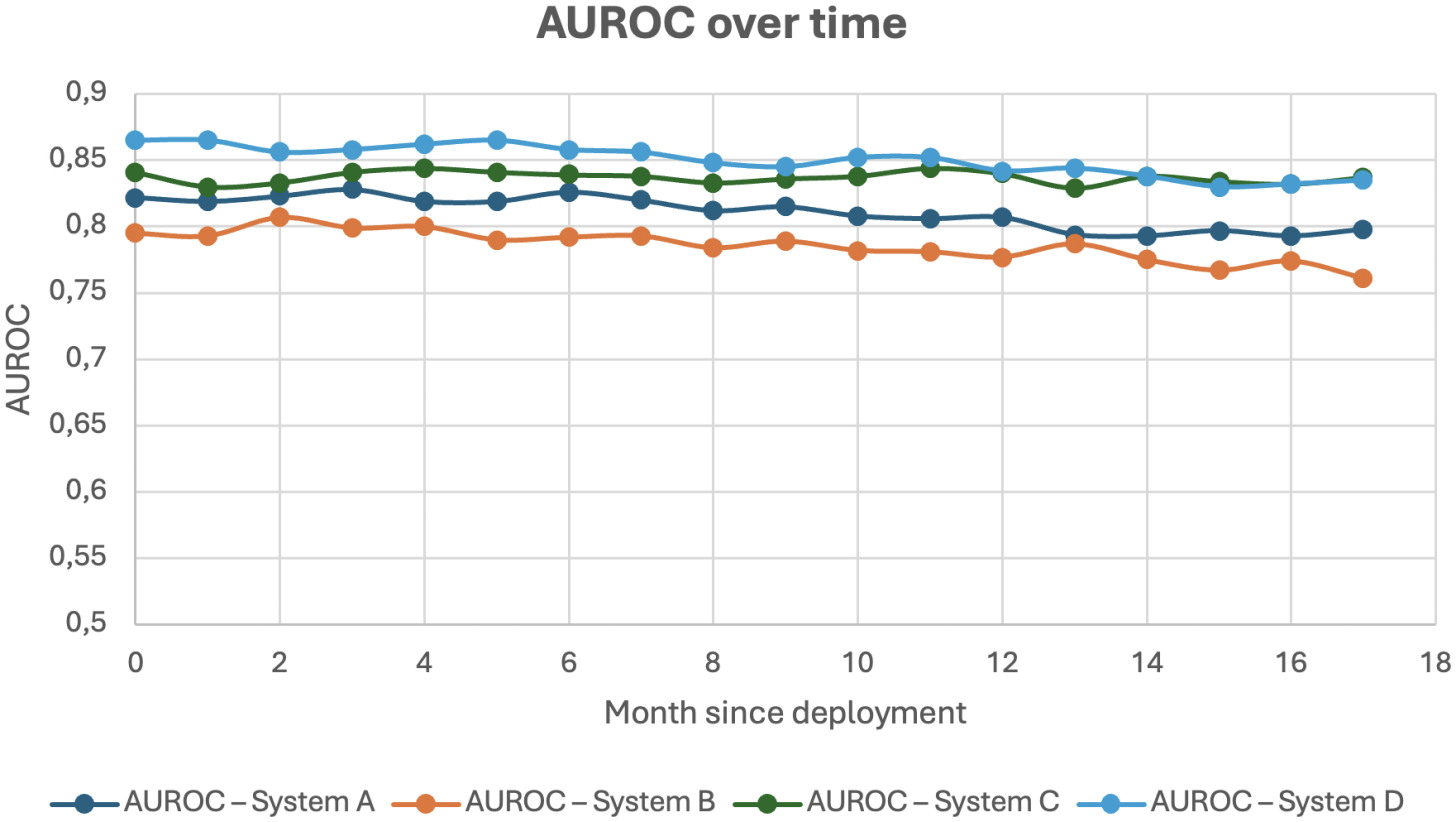
Longitudinal trajectories of predictive performance after deployment.

Performance degradation unfolded gradually rather than as abrupt shifts (Fig 1). Discrimination remained relatively stable early after deployment and declined later, whereas calibration deviated earlier and more consistently from validation benchmarks.

In Systems A and B, calibration slope attenuation and intercept drift emerged within months, preceding AUROC decline. System C showed slower calibration drift, while System D exhibited greater discrimination sensitivity to modality-specific workflow changes.

**Fig 2:**
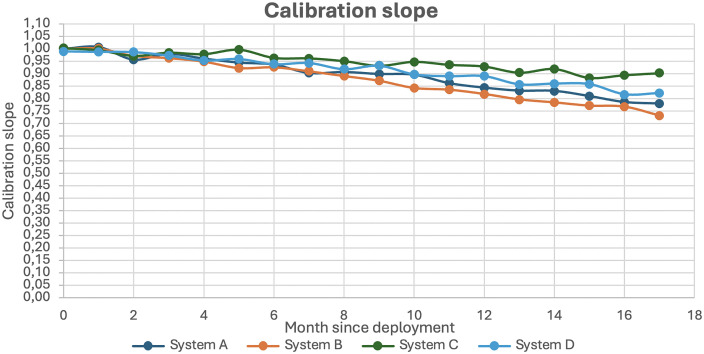
Rolling-window calibration analysis across the deployment period.

Rolling-window analyses confirmed sustained calibration divergence rather than transient instability (Fig 2). Confidence intervals widened during deployment, indicating increased performance heterogeneity under routine conditions.

**Fig 3:**
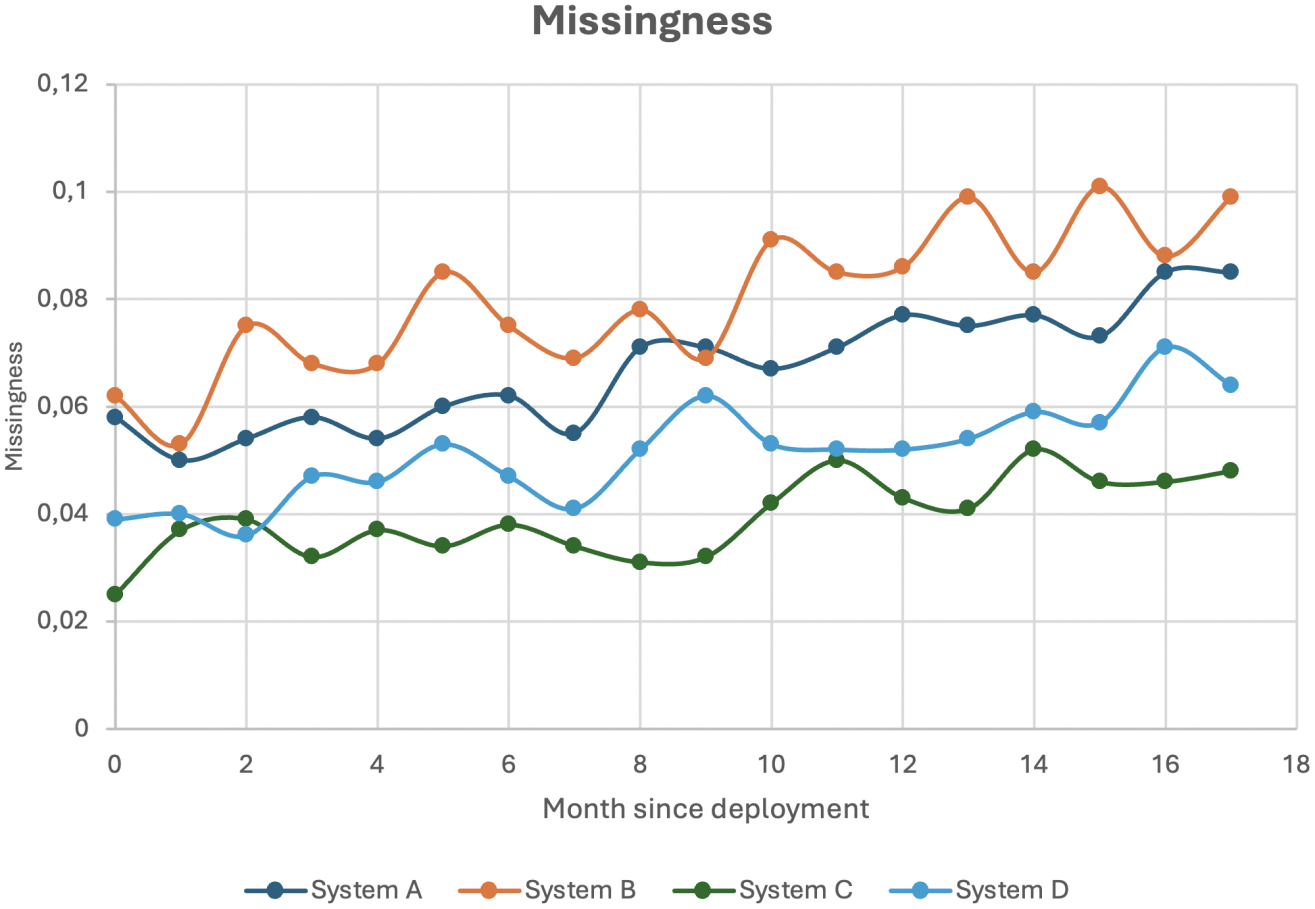
Input missingness over time.

**Fig 4:**
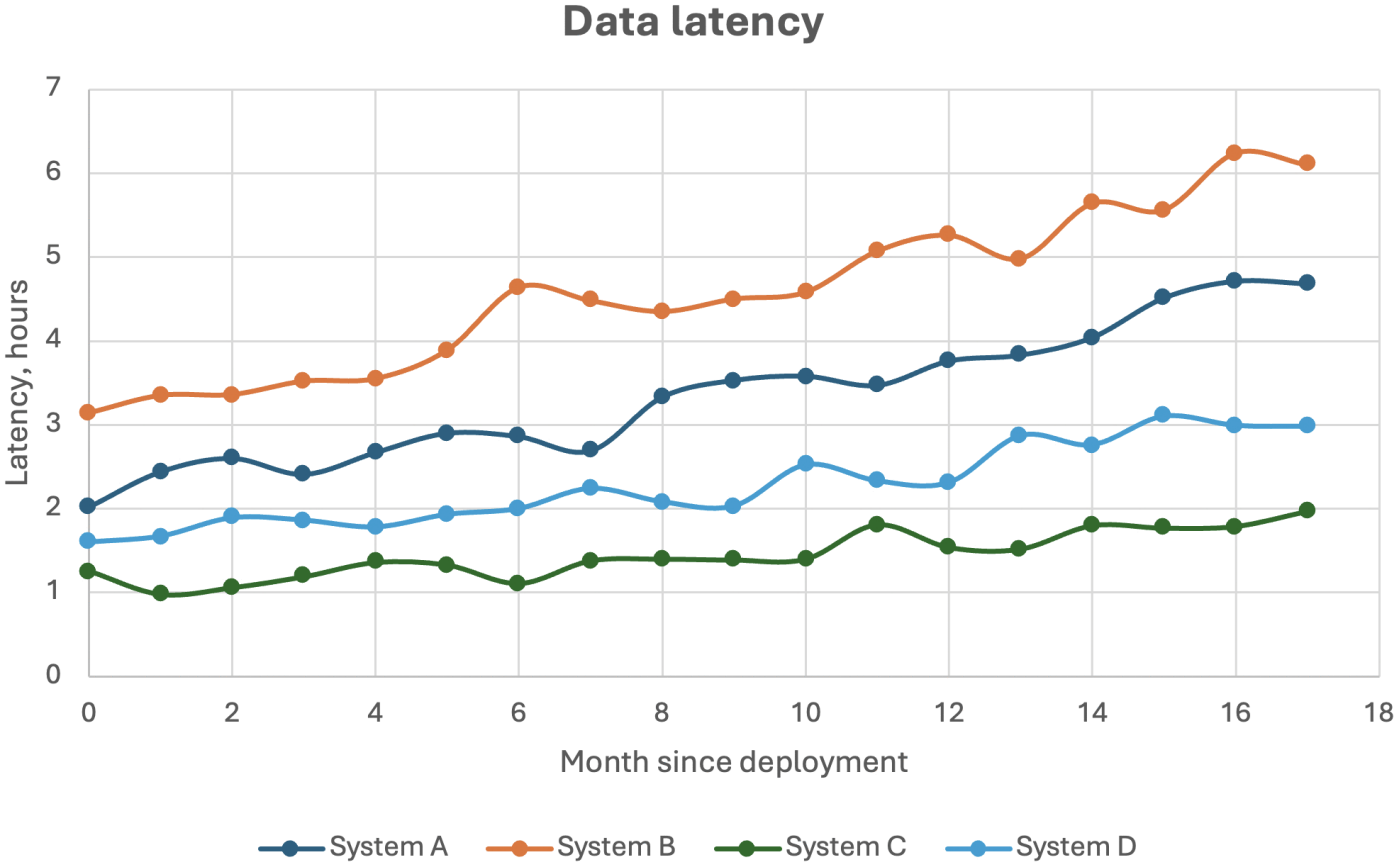
Data latency over time.

These longitudinal trajectories support the phase-level findings in Table 4 by showing that calibration degradation emerged earlier and more consistently than discrimination decline. Operational telemetry changed over the same period. Figs 2–4 show the co-evolution of missingness, latency, and calibration drift across systems.

Missingness was summarized as the proportion of expected model input fields unavailable at inference time within each post-deployment interval.

Missingness increases were not uniform across feature types. In Systems A and B, the largest increases occurred in time-sensitive EHR inputs, including recent vital-sign observations, laboratory values pending at inference time, and structured documentation fields dependent on clinical workflow completion. In Systems C and D, missingness increases were smaller but were concentrated in task-specific inputs, including delayed laboratory availability and incomplete imaging-derived feature extraction. These patterns indicate that increasing missingness reflected operational data availability and documentation timing rather than random feature loss. This supports the interpretation that missingness contributed to calibration degradation by altering the information available to the model at inference time.

Together, the telemetry and calibration trajectories indicate that operational signals were observable before or alongside performance degradation. These findings support the use of telemetry as an early monitoring signal under conditions where complete outcome labels are delayed.

### 3.5. Characterization of post-deployment failure modes

The quantitative and longitudinal analyses identified three recurrent post-deployment failure modes that would be incompletely captured by validation-era evidence alone and may be detected late under outcome-label-dependent monitoring: silent calibration failure under preserved discrimination, workflow-associated degradation without large population shift, and governance failure due to outcome-label latency. These failure modes were derived from the combined evidence in Tables 1, 4, 5 and Figs 1–4.

**Table 5:** Clinical AI systems included in the empirical evaluation.

ID	Clinical task	Data modality	Deployment setting	Prediction horizon	Inference events, thousands	Distinct patients, thousands	Outcome prevalence	Primary outcome	Typical outcome-label lag	Broad feature classes	Workflow integration mode
A	Early clinical deterioration risk prediction	EHR	Inpatient acute care	24 hours	1,240	105	8.7-9.1%	ICU transfer within 24 hours	24-72 hours	Vital signs, recent laboratory values, medication and order activity, encounter context, nursing documentation	Passive risk score and threshold-based escalation list reviewed by ward team
B	Sepsis risk stratification	EHR	Emergency department and inpatient wards	6–12 hours	410	26	6.4-7.0%	Sepsis onset based on clinical diagnosis	3-14 days	Vital signs, laboratory values, infection-related orders, medication administration, clinical documentation, prior utilization	Real-time alert integrated into emergency and ward workflow with acknowledgement/ override logging
C	Diagnostic decision support	Laboratory data and structured EHR	Specialty outpatient clinics	Diagnostic episode/ index visit	96	74	14.2-14.8%	Confirmed diagnosis	2-8 weeks	Laboratory panels, structured diagnoses, demographics, referral context, prior test history	Decision-support output available during diagnostic review; no interruptive alert
D	Outcome risk prediction	Imaging-derived features (CT/MR)	Radiology workflow	30 days	68	55	11.5-12.0%	Adverse outcome within 30 days	7-30 days	Imaging-derived quantitative features, radiology protocol metadata, demographics, selected clinical context variables	Risk estimate generated after image processing and available in radiology reporting workflow

#### 3.5.1. Failure Mode 1. Silent calibration failure under preserved discrimination.

Across systems, degradation often appeared first as calibration failure without comparable loss of discrimination. In Systems A and B, AUROC remained close to validation-era values during early deployment, while calibration slopes and intercepts had already diverged from validation estimates (Table 4; Figs 1–2).

This represents silent calibration failure: the models preserved ranking ability but systematically misestimated absolute risk. Because absolute risk estimates inform thresholds and escalation decisions, monitoring focused primarily on AUROC would delay detection of this failure mode.

Clinically, this failure mode is important because preserved ranking does not ensure correct absolute risk estimation. A model may continue to separate higher-risk from lower-risk patients while systematically overestimating or underestimating the probability of deterioration, sepsis, diagnosis, or adverse outcome. In threshold-based workflows, such miscalibration can change the number of patients crossing escalation thresholds, alter alert burden, or create false reassurance, even when AUROC remains within an apparently acceptable range.

In the present data, this pattern was most evident in Systems A and B, where early-deployment AUROC remained close to validation values, but calibration slopes and intercepts had already shifted away from validation-era estimates.

#### 3.5.2. Failure Mode 2. Workflow-associated degradation without large population shift.

A second failure mode was workflow-associated degradation without comparable population-level change. Population-related indicators showed smaller and less consistent associations with calibration degradation than workflow-associated indicators, whereas missingness, latency, and feature availability showed stronger associations across systems (Table 1).

This pattern indicates that operational changes in how data were generated, documented, or made available may destabilize deployed models even when broad population characteristics do not change to a comparable extent. The finding should be interpreted as evidence that workflow-associated telemetry was more closely aligned with degradation in this dataset, not as evidence that population change was absent or irrelevant.

#### 3.5.3. Failure Mode 3. Governance failure due to outcome-label latency.

A third failure mode was governance failure due to outcome-label latency. Operational telemetry, including missingness and latency, was observable at inference time, while outcome labels became available only after task-dependent delays ranging from days to months (Table 5; Figs 3–4).

Under outcome-based monitoring alone, degradation would therefore be detected only after a delay. This creates a governance gap in which systems may continue operating despite telemetry evidence of emerging performance degradation. In the present data, this was most relevant for Systems C and D, where outcome-label availability was delayed by weeks, while missingness, latency, and calibration trajectories were observable during deployment.

#### 3.5.4. Common structure of post-deployment failure.

Across systems, the failure modes shared a common structure: degradation was progressive, often appeared first in calibration and operational telemetry, and could remain insufficiently visible under discrimination-focused or outcome-label-dependent monitoring. This structure explains why acceptable validation performance may not provide durable assurance after deployment.

### 3.6. Sensitivity and robustness analyses

#### 3.6.1. Sensitivity to deployment phase definitions.

The primary phase-level analysis defined early deployment as the first 6 months after clinical deployment and mature deployment as the period thereafter. To evaluate whether the observed validation–deployment gap depended on this boundary, phase-level comparisons were repeated using shorter and longer early deployment definitions of 3 months and 9 months.

Across phase definitions, calibration changed more consistently than discrimination. Validation-to-early-deployment differences in calibration slope were already evident using the 3-month definition and increased modestly under the 6- and 9-month definitions. In contrast, AUROC changes during early deployment remained smaller in magnitude, supporting the interpretation that calibration degradation emerged earlier than discrimination decline (Table 6).

**Table 6:** Sensitivity analysis using alternative definitions of the early deployment phase.

	Role in analysis	Mean Δ calibration slope, validation to early deployment (95% CI)	Mean Δ AUROC, validation to early deployment (95% CI)	Direction of finding
First 3 months after deployment	Sensitivity analysis	-0.08 (-0.11 to -0.05)	-0.01 (-0.02 to 0.00)	Calibration changed earlier than discrimination
First 6 months after deployment	Primary analysis	-0.11 (-0.14 to -0.08)	-0.02 (-0.03 to -0.01)	Calibration changed earlier than discrimination
First 9 months after deployment	Sensitivity analysis	-0.14 (-0.18 to -0.10)	-0.03 (-0.05 to -0.01)	Calibration changed earlier than discrimination

The direction of the findings was unchanged across all early-deployment definitions. Calibration slope declined under each definition, with confidence intervals excluding zero, whereas AUROC differences were smaller and closer to zero. These results indicate that the main finding – earlier and more consistent calibration degradation relative to discrimination decline – was not an artifact of the selected 6-month primary phase definition.

#### 3.6.2. Sensitivity to temporal aggregation and windowing.

Longitudinal estimates were repeated using alternative temporal aggregation windows. Shorter windows increased variability, especially in lower-volume systems, whereas wider windows reduced short-term fluctuation. The direction and timing of the main findings were preserved across windowing specifications.

Calibration degradation remained detectable earlier than discrimination decline across alternative aggregation windows. Workflow-associated telemetry signals, particularly missingness and latency, continued to precede or co-evolve with calibration degradation. These analyses indicate that the observed longitudinal patterns were not artifacts of the selected temporal window.

#### 3.6.3. System-level exclusion and heterogeneity analyses.

Leave-one-system-out analyses preserved the direction and ranking of associations between workflow-associated indicators and calibration degradation. Missingness and latency remained negatively associated with calibration slope in all exclusions, and workflow-associated indicators retained greater explanatory contribution than population-related indicators (Table 2).

In all leave-one-system-out analyses, workflow-associated indicators retained stronger associations with calibration degradation than population-related indicators. The temporal ordering between telemetry change and subsequent performance degradation was also preserved.

#### 3.6.4. Robustness of telemetry–performance associations.

Associations between operational telemetry and calibration degradation were re-estimated using alternative regression specifications, including alternative covariate sets and exclusion of potentially collinear predictors (Table 3).

Input missingness and data latency preserved negative associations with calibration slope in all tested specifications. Feature availability change preserved the expected direction in most specifications, whereas population demographics showed a small coefficient range, lower incremental explained variance, and inconsistent direction. These findings indicate that the telemetry–performance relationship was not driven by a single regression specification.

#### 3.6.5. Summary of robustness findings.

Across sensitivity and robustness analyses, the main findings were unchanged: validation-era performance did not persist as a stable operational property after deployment; calibration degraded earlier and more consistently than discrimination; workflow-associated indicators showed stronger associations with degradation than population-related indicators; and operational telemetry provided earlier observable signals than outcome-based reassessment alone.

## 4. Discussion

### 4.1. Principal findings and conceptual contribution

This study provides empirical, longitudinal evidence that post-deployment fragility can arise systematically across clinical AI systems operating under routine conditions.

#### 4.1.1. Principal finding 1. Validation performance does not persist as an operational property of deployed systems.

Across all systems, models that met validation criteria diverged materially from their validation-era performance after deployment despite unchanged parameters, features, and intended use. This extends critiques of retrospective validation by showing the validation–deployment gap arises within a single institution over time, not only through cross-site transportability failures [15,33]. Validation should therefore be treated as a conditional snapshot tied to the historical workflow, documentation, and data-pipeline context, challenging the assumption in reporting and procurement that acceptable validation metrics bound future operational risk.

#### 4.1.2. Principal finding 2. Calibration drift is an early, dominant, and clinically silent failure mode.

Calibration degradation consistently preceded and exceeded discrimination loss after deployment: across multiple systems, slopes and intercepts drifted substantially while AUROC remained acceptable for extended periods. This extends prior reports of temporal miscalibration [32–34] by showing calibration failure is structural and progressive rather than sporadic. Silent miscalibration is therefore a first-order safety risk, as it distorts absolute risk estimates and threshold-based actions without triggering discrimination-focused alarms, highlighting a gap between commonly reported metrics and clinically meaningful safety properties.

#### 4.1.3. Principal finding 3. Workflow-associated indicators were more strongly associated with degradation than population-related indicators.

Workflow-associated and operational telemetry signals showed stronger and more consistent associations with post-deployment calibration degradation than population-related indicators. Across systems, rising input missingness, data latency, and feature availability shifts aligned with – and often preceded – calibration degradation, whereas demographic and outcome-prevalence indicators showed smaller and less consistent associations. This supports a workflow-aware interpretation of post-deployment fragility: degradation may be linked to how data are produced within evolving workflows and information systems, not only to who the patients are [8,18,33,35].

Together, these results frame clinical AI fragility as an operational property of deployed systems under real governance constraints (e.g., delayed/biased labels). The study shifts emphasis from cross-site transportability toward within-site temporal fragility and motivates telemetry-informed, calibration-aware monitoring over outcome-centric audits as the basis for lifecycle assurance.

### 4.2. Comparison with state-of-the-art deployed systems and contribution of the present study

Real-world evidence on clinical AI has expanded beyond retrospective validation to external audits, prospective deployments, and workflow-level evaluations. However, as summarized in Table 7, it remains fragmented regarding when degradation begins, which signals detect it, and how governance constraints such as label latency limit observability. The present study addresses this gap directly.

**Table 7:** Deployed clinical AI systems and governance-relevant observability.

Deployed clinical AI system	Task and clinical domain	Real-world evaluation	Labels available post-deployment	Available label-independent monitoring signals
Epic Systems Epic Sepsis Model (ESM)	Sepsis prediction (EHR)	External validation showing poor transportability/ calibration issues [15]	Sepsis labels typically **days–weeks** (coding/ chart review), mortality **days,** ICU transfer **hours-days** but policy-dependent.	Missingness, timestamp/latency, feature availability drift, alert firing rates; CDS logs: acknowledgement, time-to-action.
Epic Systems Epic Deterioration Index (EDI)	In-hospital deterioration risk (EHR)	Independent evaluation in COVID-19 admissions [36]	Composite deterioration outcomes **hours-days**; mortality **days,** labeling sensitive to capacity thresholds.	Missingness, documentation frequency, latency, score distribution drift, escalation timing.
TREWS	Sepsis early warning + provider interaction	Prospective multi-site evaluation [37,38]	Sepsis labels **days-weeks,** treatment timing **minutes-hours**.	Alert acknowledgement, override, time-to-evaluation, time-to-antibiotics, alert volume per unit.
IDx-DR	Autonomous diabetic retinopathy screening	Pivotal prospective trial [39]	Reference grading **days-weeks,** referral outcomes **months**.	Image quality/ gradability, device metadata, failure-to-grade, site-level drift.
Eyenuk EyeArt	Autonomous diabetic retinopathy screening	Pivotal evaluation [40]	Adjudicated DR grade **days-weeks,** downstream outcomes **months**.	Gradability flags, operator/device effects, turnaround times, referral conversion.
Viz.ai Viz LVO	LVO detection on CTA	Real-world evaluation in stroke center [41]	Final neuroradiology read **hours-days**; reperfusion outcomes **days**.	Scan-to-alert latency, false alert rate, protocol drift, user open/ack logs.
RapidAI	Stroke triage workflow acceleration	Real-world association with faster treatment [42]	Procedural timestamps **minutes–hours,** outcomes **days-months**.	Notification delivery, latency, user engagement, team activation timing, pipeline uptime.
Thailand national DR screening	Population-scale DR screening	Prospective program + deployment analysis [43,44]	Specialist grading **days-weeks,** programmatic outcomes **months**.	Image gradability, queue delays, operator/site heterogeneity, data integrity failures.
**Present study**	Multi-task, multi-modality clinical AI	Methodological longitudinal analysis	Labels delayed **days-months**, task-dependent, assumed unavailable for real-time safety monitoring.	Explicitly modeled: missingness, latency, feature availability change, distribution shift statistics, governance-relevant telemetry.

#### 4.2.1. What existing deployment evidence establishes.

Table 7 shows that deployment evidence consistently highlights context sensitivity, calibration limitations, and workflow dependence across clinical AI. External audits (e.g., ESM, EDI) demonstrate that validation performance does not guarantee acceptable real-world behavior, while prospective and workflow-oriented studies (e.g., TREWS, RapidAI) indicate that clinician interaction and operational integration materially shape outcomes.

Table 7 also underscores a common constraint: outcome labels rarely arrive fast enough for continuous safety assurance. Diagnostic confirmation, sepsis adjudication, and specialist grading are typically delayed days to weeks, and downstream outcomes may lag months, so evaluations often identify failures retrospectively rather than enabling early detection.

#### 4.2.2. What remains missing in the state of the art.

Despite the breadth of systems represented in Table 7, prior work generally does not address three interrelated questions that are central to governance:

1. **When does degradation begin relative to deployment?**

Most studies assess performance at isolated time points, obscuring early-stage divergence from validation behavior.

2. **Which observable signals precede outcome-measured failure?**

Although telemetry such as missingness, latency, acknowledgement, and image quality is routinely available, it is rarely analyzed as a predictor of safety-relevant degradation.

3. **How do governance constraints shape observability?**

Label latency and policy-dependent outcomes are acknowledged but seldom incorporated into evaluation design.

#### 4.2.3. Contribution of the present study relative to deployed systems.

The present study addresses these gaps by using longitudinal, telemetry-linked analysis to characterize post-deployment fragility under realistic governance constraints. The analysis is explicitly designed to mirror the observability conditions documented in Table 7 and to extract generalizable failure structures while grounding them in empirical effect magnitudes observed under routine deployment conditions.

Relative to existing deployment evidence, the contribution of this study is threefold:

- It unifies disparate observations from prior audits and deployments into a coherent set of post-deployment failure modes (Section 3.5).
- It demonstrates that label-independent operational telemetry can provide earlier indication of degradation than outcome-based reassessment when labels are delayed.
- It reframes many observed breakdowns as governance failures – cases where degradation is detectable in principle but invisible under prevailing monitoring practices.

By positioning the present study alongside real deployed systems in Table 7, the analysis clarifies that the central challenge is not lack of data, but lack of conceptual and operational alignment between validation, monitoring, and governance.

### 4.3. Implications for post-deployment clinical AI evaluation and governance

The findings of this study, derived from longitudinal analysis of real-world clinical AI deployments and considered alongside prior deployment evidence summarized in Table 7, reveal structural limitations of validation-centric approaches to post-deployment safety assurance. These implications extend beyond technical model assessment and speak directly to the design of lifecycle assurance frameworks that are credible under real-world clinical constraints.

#### 4.3.1. Reframing validation as conditional evidence, not proof of safety.

A central implication is that validation should be interpreted as conditional and time-bound evidence, rather than as proof of durable clinical safety. Current practice often treats acceptable discrimination and calibration at validation as sufficient justification for deployment, with the implicit assumption that performance will remain stable unless the model is explicitly updated. The longitudinal degradation observed in this study – and mirrored by failures documented in real deployments – shows that this assumption does not hold.

Importantly, this implication does not depend on precise effect sizes, but on the recurrent structure and temporal ordering of degradation observed across empirical deployments and reproduced in the present analysis.

From a governance perspective, validation should therefore be repositioned as establishing a baseline operating envelope rather than a safety guarantee. This aligns with emerging regulatory language emphasizing lifecycle management but requires a more explicit translation into operational policy: deployment decisions should include predefined expectations about monitoring, reassessment frequency, and criteria for intervention when post-deployment behavior diverges from validated performance.

#### 4.3.2. Elevating calibration stability to safety objective.

The consistent emergence of calibration drift as an early failure mode suggests that calibration stability should be treated as a primary safety objective, rather than a secondary reporting metric. While discrimination remains important for ranking tasks, calibration governs the correctness of absolute risk estimates that underpin threshold-based decisions, escalation pathways, and clinical trust.

In practical terms, this implies that post-deployment monitoring frameworks should include explicit calibration checks – such as rolling calibration slopes or intercepts – rather than relying solely on AUROC or alert counts. Importantly, these checks need not depend on immediate outcome availability; partial labeling, delayed outcomes, or proxy events can still support periodic calibration assessment if designed deliberately. Without such emphasis, systems may remain in use during prolonged periods of silent miscalibration.

#### 4.3.3. From population drift to workflow-aware monitoring.

A further implication concerns the importance of workflow-associated telemetry. Much existing guidance implicitly prioritizes monitoring for population drift – changes in demographics, case mix, or disease prevalence. While relevant, the results of this study indicate that workflow-aware signals were more consistently associated with degradation and may be more actionable for monitoring.

Governance frameworks should therefore explicitly incorporate operational telemetry – input missingness, data latency, feature availability, documentation frequency, and interaction logs – as monitoring signals. These data are already collected by most clinical systems, as shown in Table 7, but are rarely analyzed in relation to model safety. Integrating such signals into routine monitoring shifts the focus from abstract statistical change to operational integrity, which is both observable in real time and more directly controllable by healthcare organizations.

These findings support a telemetry-first monitoring approach in which operational indicators are used for early warning and triage, while outcome-based reassessment serves as confirmatory evidence once labels become available. Although the present study focused on EHR-, laboratory-, and imaging-derived systems, the same governance logic may extend to omics-based clinical AI models. Omics inputs are not passively observed biological facts; they are generated through laboratory and bioinformatic workflows involving sample handling, sequencing or assay platforms, reagent lots, read depth or coverage, normalization, batch correction, and pipeline versions. Recent omics-based diagnostic models illustrate both the promise of transferring single-cell or molecular signatures across bulk and single-cell sequencing data and the dependence of such models on platform compatibility, normalization, and cohort-specific processing choices [45–47]. In this context, telemetry-first monitoring would need to include technical telemetry such as sample quality metrics, batch identifiers, assay or sequencing platform version, coverage or read-depth indicators, normalization parameters, and bioinformatic pipeline provenance. These signals may serve an analogous role to EHR missingness and latency by identifying changes in the data-generation process before outcome labels or clinical endpoints are available. The operational implication of this approach is summarized in Table 8, which translates the observed sequence of telemetry change, performance degradation, delayed labels, and governance response into a lifecycle monitoring framework.

**Table 8:** Telemetry-first lifecycle governance framework for deployed clinical AI.

Lifecycle stage	Primary purpose	What is evaluated/ observed	Typical data availability	Governance-relevant decisions
Pre-deployment validation	Establish baseline operating envelope	Discrimination, calibration, subgroup performance under historical conditions	Fully labeled retrospective data	Approve deployment *conditional* on monitoring and reassessment plan
Initial deployment	Integrate model into clinical workflow	Model outputs, workflow integration, data pipeline integrity	Immediate (real-time inference)	Confirm technical integration, validate assumptions about data availability
Continuous telemetry monitoring	Detect early signals of degradation	Input missingness, data latency, feature availability, score distribution drift, interaction logs	Real time/ near real time	Trigger investigation before outcome labels are available
Drift detection and triage	Assess safety relevance of observed changes	Temporal trends in telemetry, early calibration proxies, workflow changes	Days–weeks	Decide whether degradation is benign, workflow-induced, or safety-relevant
Governance action	Mitigate risk	Threshold adjustment, workflow correction, recalibration, model suspension	Before outcome confirmation	Prevent continued silent failure during label delay
Outcome-based reassessment	Confirm magnitude and impact of degradation	Calibration and discrimination using delayed outcome labels	Days–months	Validate prior actions; update risk assessment
Reassessment and adjustment	Restore or retire system	Updated performance metrics; revised operating envelope	Episodic	Retrain, redeploy, or decommission model
Ongoing operation	Maintain lifecycle assurance	Combined telemetry + periodic outcome review	Continuous	Sustain safe use under evolving conditions

#### 4.3.4. Designing monitoring under label latency constraints.

Perhaps the most consequential implication is the need to design monitoring strategies that are robust to label latency and label bias. As summarized in Table 7, real-world deployed clinical AI systems commonly rely on outcome labels that become available only after task-dependent delays ranging from hours or days to weeks or months. This delay makes outcome-based monitoring insufficient as the sole mechanism for timely post-deployment safety surveillance.

The present study supports a telemetry-first monitoring paradigm, in which label-independent signals are used for early warning and triage, while outcome-based reassessment serves as confirmatory evidence rather than the sole trigger for action. This does not eliminate the need for outcome evaluation, but it changes its role within the governance lifecycle. Such an approach aligns more closely with how safety is managed in other high-reliability systems, where indirect indicators of system health are used to prevent failures before harm occurs.

A related concern is label feedback. Once a deployed AI system influences clinical decisions, subsequent outcome labels may partly reflect model-guided intervention rather than the unmodified natural history of disease. For example, an early warning score that triggers earlier treatment may change the later outcome used to audit the model. This can bias outcome-dependent reassessment and make delayed labels difficult to interpret as independent ground truth. Telemetry-first monitoring does not solve this causal problem, but it reduces dependence on potentially contaminated outcome labels for early safety surveillance. Signals such as input missingness, data latency, feature availability, score-distribution change, and interaction logs are observable at or near inference time, before downstream clinical response is fully expressed. They should therefore be interpreted not as unbiased measures of clinical effectiveness, but as less label-dependent indicators of data-pipeline integrity, workflow stability, and changing operating conditions.

#### 4.3.5. Implications for organizational accountability and actionability.

Finally, reframing post-deployment fragility as a governance problem has implications for accountability. If degradation is driven by workflow evolution, documentation changes, or data pipeline failures, then responsibility for safety cannot reside solely with model developers. Instead, accountability must be distributed across clinical operations, information technology, and quality governance functions.

Monitoring outputs should be actionable, meaning that they are linked to predefined responses such as investigation, recalibration, workflow adjustment, threshold review, or temporary suspension. Without such linkages, even sophisticated monitoring risks becoming descriptive rather than protective. Deployment decisions should therefore be accompanied by explicit expectations about how performance may change over time, predefined monitoring plans linked to validated metrics, and criteria for reassessment or intervention when deviations occur. By identifying which signals are both observable and associated with degradation, this study provides a foundation for making post-deployment governance operational rather than aspirational.

### 4.4. Limitations and scope of interpretation

Several limitations should be considered when interpreting the findings of this study. These limitations primarily relate to the methodological scope, the use of longitudinal analyses, and the intentional abstraction from specific clinical implementations, and they delineate how the results should be used and not used.

#### 4.4.1. Scope of empirical evidence and generalizability.

While this study is grounded in real-world deployment data, the empirical scope is limited to a finite set of clinical AI systems operating within specific institutional contexts. As a result, quantitative effect sizes and degradation rates should not be interpreted as universal. However, the consistency of observed failure structures across heterogeneous systems supports the broader generalizability of the qualitative findings regarding timing, observability, and governance relevance of post-deployment degradation.

#### 4.4.2. Abstraction across heterogeneous clinical tasks and modalities.

The study intentionally abstracts across multiple clinical tasks and data modalities. While this enables identification of cross-cutting failure modes, it necessarily simplifies task-specific nuances. For example, the clinical consequences of calibration drift differ between early warning systems, autonomous diagnostic tools, and workflow acceleration platforms. Similarly, the availability and reliability of telemetry signals may vary by modality and vendor implementation.

For omics-based models, relevant telemetry may include assay platform, reagent batch, sample quality, sequencing depth, normalization procedures, batch-correction methods, and bioinformatic pipeline version; these were outside the empirical scope of the present analysis.

As a result, the findings should be interpreted as conceptual guidance for evaluation and governance, rather than as prescriptive thresholds applicable to all clinical AI systems. Institutions applying these insights should tailor monitoring strategies to the specific clinical context, decision pathways, and risk tolerance of each deployment.

#### 4.4.3. Lack of prospectively elicited clinician tolerance thresholds.

The study did not include prospectively elicited clinician tolerance thresholds for performance degradation or direct measures of clinician trust, reliance, or abandonment over time. Therefore, we did not estimate system-specific time-to-loss-of-trust thresholds. The clinical relevance of calibration drift was interpreted through its effect on absolute risk estimation and threshold-based decision-making rather than through measured clinician acceptability. Future prospective studies should define task-specific tolerance thresholds with clinical users and evaluate how calibration and discrimination changes affect trust, reliance, and continued system use.

#### 4.4.4. Limited representation of organizational response and mitigation actions.

The analyses focus on detection and characterization of degradation rather than on organizational responses once degradation is identified. In real deployments, health systems may intervene through recalibration, workflow modification, retraining, or temporary suspension, potentially altering observed trajectories. In addition, model-generated predictions may influence clinician behavior and subsequent outcome labels, creating label feedback that complicates outcome-based reassessment. These adaptive responses and feedback mechanisms were not modeled explicitly.

This limitation reflects a broader gap in the literature: few published studies document not only when degradation occurs, but also how organizations respond and with what effectiveness. Future work should examine mitigation strategies as objects of study, integrating technical, clinical, and governance perspectives.

#### 4.4.5. Generalizability to regulatory decision-making.

Finally, while the study engages with regulatory and governance concepts, it does not attempt to evaluate compliance with specific regulatory frameworks or to prescribe regulatory thresholds.

The findings are relevant for organizational governance and quality assurance, particularly in informing how monitoring systems might be designed to align with lifecycle-oriented regulatory expectations. The study should therefore be viewed as contributing to the operationalization of lifecycle principles rather than as evidence for regulatory approval decisions.

Taken together, these limitations underscore that the present work should be interpreted as a conceptual and methodological contribution grounded in realistic deployment conditions, rather than as a definitive empirical evaluation of specific clinical AI systems. Its value lies in articulating how and why validated models may fail silently after deployment, and in identifying observability gaps that governance frameworks must address to ensure clinical safety over time.

## 5. Conclusions

This longitudinal analysis of four deployed clinical AI systems shows that acceptable validation performance did not persist as a stable operational property after deployment. Across systems, degradation was progressive, often appeared first as calibration drift, and was more consistently associated with workflow-related telemetry signals such as missingness, latency, and feature availability than with broad population-level indicators. These findings support treating validation as a baseline operating reference rather than a durable safety guarantee. Post-deployment governance should therefore include continuous, calibration-aware monitoring and operational telemetry review, particularly when outcome labels are delayed or incomplete.
