# Supplementary material for: Validation is not enough: Longitudinal evidence of post-deployment fragility in clinical AI systems
Source: PLOS Digit Health. 2026 Jul 27;5(7):e0001534. doi: 10.1371/journal.pdig.0001534 (PMC13405297; doi:10.1371/journal.pdig.0001534)
Supplement: Attachment — Submitted filename: response PLOS.docx [file pdig.0001534.s001.docx]

Reviewer #1:

**1. Methods: The methods tend to make general/generic statements and require more specificity to clearly describe the planned analyses reported in the results. For example, the results report sensitivity analysis on temporal aggregation, but this is not mentioned in the methods. For the reader, this creates a discordance between expected analyses and actual analyses that was reported.**

We standardized the terminology for deployment phases and revised the Methods to define the primary phase boundaries explicitly. In the revised manuscript, early deployment is defined as the first 6 months after deployment, and mature deployment as the period thereafter. We also clarified that 3- and 9-month early deployment definitions were used as sensitivity analyses to assess whether the findings depended on the selected phase boundary.

Statistical analyses were designed to quantify longitudinal divergence between validation-era and post-deployment model behavior and to examine whether this divergence was associated with temporal, population-related, or workflow-associated indicators [30,31].

All analyses were performed separately for each AI system and, where specified, in pooled models including all systems.

The individual inference event was the base unit of analysis. For longitudinal analyses, inference events were aggregated by system and post-deployment time interval. Monthly aggregation was used for the main longitudinal trajectories. Phase-level analyses compared validation, early deployment, and mature deployment periods.

For each system and time interval, we calculated AUROC, AUPRC where applicable, calibration slope, calibration intercept, input missingness, data latency, feature availability, inference volume, and available system-interaction measures. Confidence intervals for performance metrics were estimated using bootstrap resampling within each system and time interval.

Longitudinal performance trajectories were summarized using monthly estimates and rolling-window analyses. Sensitivity analyses repeated the longitudinal analyses using alternative temporal aggregation windows to assess whether the observed direction and timing of performance degradation depended on the selected window size.

Deployment-phase comparisons used the primary validation, early deployment, and mature deployment definitions specified in Section 2.1. Sensitivity analyses repeated the phase-level comparisons using alternative early deployment definitions of 3 and 9 months, with the 6-month definition used as the primary analysis.

Regression analyses were performed at the system-time-interval level, where each observation represented one AI system within one post-deployment temporal interval. The primary dependent variable for drift-association analyses was calibration slope, because calibration degradation was the earliest and most consistent post-deployment failure pattern. Secondary dependent variables were calibration intercept, AUROC, and AUPRC where applicable.

Population-related indicators included demographic composition, available case-mix variables, and outcome prevalence. Workflow-associated indicators included input missingness, data latency, feature availability, inference volume, and available interaction measures. Continuous covariates were standardized before regression modelling.

The primary pooled model used a mixed-effects linear regression with a system-level random intercept. The general model form was:

*Performanceₛ,ₜ = β₀ + β₁Timeₛ,ₜ + β₂PopulationIndicatorsₛ,ₜ + β₃WorkflowIndicatorsₛ,ₜ + uₛ + εₛ,ₜ ,ß*

where *s* denotes the AI system, *t* denotes the post-deployment time interval, *u*s is the system-level random intercept, and εs,t is the residual error.

To compare population-related and workflow-associated indicators, three model blocks were estimated: a population-only block, a workflow-only block, and a combined population-plus-workflow block. The relative contribution of each block was summarized using standardized regression coefficients, 95% confidence intervals, changes in explained variance, and consistency of effect direction across systems and robustness specifications. These analyses were interpreted as associations between covariate blocks and performance degradation, not as causal decomposition.

Lagged analyses related operational telemetry indicators from preceding time intervals to subsequent performance metrics. These analyses used the same dependent variables and model structure as the primary regression analyses.

obustness analyses included leave-one-system-out models and alternative covariate specifications. For alternative regression specifications, robustness was summarized using the range of standardized regression coefficients across specifications, the proportion of specifications preserving the expected direction of association, and the median incremental explained variance associated with each signal category. Sensitivity analyses included alternative deployment-phase definitions, alternative temporal aggregation windows, and alternative regression specifications.

Results were summarized using effect estimates, 95% confidence intervals, explained-variance changes, and consistency across systems and robustness specifications.

**2. Methods: Manuscript states “Analyses were conducted using standard statistical software with version-controlled scripts to ensure reproducibility.” Please provide more detail on the statistical software package used. Also, consider making your code publicly available along with the manuscript for transparency of the statistical analysis.**

We revised the Methods section to specify the statistical software environment, including Python and package versions used for data handling, metric estimation, regression modelling, bootstrapping, sensitivity analyses, and figure generation. We also added a Code Availability statement clarifying that analysis scripts and aggregate reproducibility materials will be made available, while patient-level clinical and operational data cannot be publicly released.

Analyses were performed in Python 3.11.7. Data processing and temporal aggregation were performed using pandas 2.1.4 and NumPy 1.26.4. Performance metrics, including AUROC and AUPRC, were calculated using scikit-learn 1.3.2. Calibration slope and calibration intercept were estimated using statsmodels 0.14.1. Bootstrap confidence intervals, regression analyses, lagged analyses, sensitivity analyses, and leave-one-system-out robustness analyses were implemented using NumPy 1.26.4, pandas 2.1.4, scikit-learn 1.3.2, statsmodels 0.14.1, and SciPy 1.11.4. Figures were generated using matplotlib 3.8.2. All scripts were maintained under version control.

**3. Results report different temporal periods or phases e.g. “Early Phase” or “Early Deployment”, etc. but it is unclear how these temporal phases are defined. Please standardize the definition for these and clearly define them and the principles by which they were chosen as well as justification to why your definitions are appropriate as opposed to some other arbitrary temporal phase definition.**

We standardized the terminology to “early deployment” and “mature deployment” throughout the manuscript. We revised the Methods to define the primary phase boundaries explicitly and added sensitivity analyses using alternative 3-, 6-, and 9-month early deployment definitions to show that the main findings were not dependent on a single temporal cut-point.

Revised **Study phases and unit of analysis** section:

For each AI system, the evaluation period was divided into three analytically distinct phases.

- **The validation phase** corresponded to the dataset and time window used for internal or temporal validation before deployment.
- **The early deployment phase** was defined in the primary analysis as the first 6 months after clinical deployment, corresponding to the initial post-integration period during which workflows and data pipelines were expected to stabilize.
- **The mature deployment** phase was defined as the period after the first 6 months of deployment, corresponding to sustained routine operation.

To assess whether the findings depended on this phase boundary, sensitivity analyses repeated the phase-level comparisons using alternative early deployment definitions of 3 months and 9 months. These analyses were used to evaluate the robustness of the validation–deployment comparison to shorter and longer assumptions about the initial post-integration period.

The primary unit of analysis was the individual inference event, defined as a single execution of the AI system producing a prediction for a specific patient encounter. Longitudinal analyses were performed using temporally aggregated system-level estimates.

Section 3.6.1 was revised to:

The primary phase-level analysis defined early deployment as the first 6 months after clinical deployment and mature deployment as the period thereafter. To evaluate whether the observed validation–deployment gap depended on this boundary, phase-level comparisons were repeated using shorter and longer early deployment definitions of 3 months and 9 months.

Across phase definitions, calibration changed more consistently than discrimination. Validation-to-early-deployment differences in calibration slope were already evident using the 3-month definition and increased modestly under the 6- and 9-month definitions. In contrast, AUROC changes during early deployment remained smaller in magnitude, supporting the interpretation that calibration degradation emerged earlier than discrimination decline (Table 4).

Table 4 was revised to

**Table 4. Sensitivity analysis using alternative definitions of the early deployment phase**

|  | Role in analysis | Mean Δ calibration slope, validation to early deployment (95% CI) | Mean Δ AUROC, validation to early deployment (95% CI) | Direction of finding |
| --- | --- | --- | --- | --- |
| First 3 months after deployment | Sensitivity analysis | -0.08 (-0.11 to -0.05) | -0.01 (-0.02 to 0.00) | Calibration changed earlier than discrimination |
| First 6 months after deployment | Primary analysis | -0.11 (-0.14 to -0.08) | -0.02 (-0.03 to -0.01) | Calibration changed earlier than discrimination |
| First 9 months after deployment | Sensitivity analysis | -0.14 (-0.18 to -0.10) | -0.03 (-0.05 to -0.01) | Calibration changed earlier than discrimination |

**4. Table 2: Please provide temporal range definitions for “Early Deployment” vs. “Mature Deployment”**

We revised Table 2 to specify the temporal ranges used for deployment phases. The caption now states that early deployment refers to the first 6 months after deployment and mature deployment refers to the subsequent observation period. This definition is consistent with the revised Methods and with the sensitivity analyses reported in Table 4.

| **System ID** | **Phase** | **Temporal definition** | **AUROC** | **AUPRC** | **Calibration slope** | **Calibration intercept** |
| --- | --- | --- | --- | --- | --- | --- |
| System A | Validation | Pre-deployment validation dataset/time window | 0.82 (0.81–0.83) | 0.38 (0.36–0.40) | 1.01 (0.97–1.05) | 0.02 (-0.01 – 0.05) |
|  | Early deployment | Months 0–5 after deployment | 0.81 (0.79–0.82) | 0.36 (0.34–0.38) | 0.88 (0.82–0.94) | -0.18 (-0.24 – -0.12) |
|  | Mature deployment | Month 6 onward | 0.78 (0.76–0.80) | 0.32 (0.30–0.34) | 0.71 (0.65–0.77) | -0.36 (-0.42 – -0.30) |
| System B | Validation | Pre-deployment validation dataset/time window | 0.80 (0.78–0.82) | 0.29 (0.27–0.31) | 0.99 (0.93–1.05) | 0.01 (-0.04 –0.06) |
|  | Early deployment | Months 0–5 after deployment | 0.79 (0.77–0.81) | 0.27 (0.25–0.29) | 0.86 (0.79–0.93) | -0.21 (-0.28 – -0.14) |
|  | Mature deployment | Month 6 onward | 0.75 (0.73–0.77) | 0.24 (0.22–0.26) | 0.69 (0.62–0.76) | -0.41 (-0.48 – -0.34) |
| System C | Validation | Pre-deployment validation dataset/time window | 0.84 (0.82–0.86) | 0.42 (0.39–0.45) | 1.02 (0.96–1.08) | -0.01 (-0.05 –0.03) |
|  | Early deployment | Months 0–5 after deployment | 0.83 (0.80–0.85) | 0.41 (0.38–0.44) | 0.93 (0.86–1.00) | -0.09 (-0.15 – -0.03) |
|  | Mature deployment | Month 6 onward | 0.82 (0.79–0.84) | 0.40 (0.37–0.43) | 0.85 (0.78–0.92) | -0.17 (-0.23 – -0.11) |
| System D | Validation | Pre-deployment validation dataset/time window | 0.86 (0.84–0.88) | 0.46 (0.43–0.49) | 1.03 (0.97–1.09) | 0.00 (-0.04 –0.04) |
|  | Early deployment | Months 0–5 after deployment | 0.83 (0.81–0.85) | 0.43 (0.40–0.46) | 0.95 (0.88–1.02) | -0.08 (-0.14 – -0.02) |
|  | Mature deployment | Month 6 onward | 0.79 (0.77–0.81) | 0.39 (0.36–0.42) | 0.89 (0.82–0.96) | -0.19 (-0.25 – -0.13) |

**5. Table 3: Please provide a caption or table legend that describes the definition of “Weak”, “Moderate”, “Strong”, and “Not Applicable”. Are these standardized effect sizes? Please describe in methods how these values are determined–there is currently a high-level but vague description. Please describe actual statistical methods, formulas, thresholds, definitions.**

We revised Table 3 to replace qualitative categories with quantitative association estimates. The revised table reports standardized regression coefficients with 95% confidence intervals for the association between candidate drift indicators and calibration slope. We also revised the Methods to specify that continuous drift indicators were standardized before model fitting and that calibration slope was used as the primary dependent variable for this analysis.

Associations between candidate drift indicators and calibration degradation are summarized in Table 3. Workflow-associated indicators showed larger and more consistent negative associations with calibration slope than population-related indicators. Input missingness and data latency were most strongly associated with declining calibration slope in Systems A and B. Feature availability change showed the strongest association in Systems B and D. Population demographics showed weak and inconsistent associations across all systems, while outcome prevalence showed modest associations in Systems A and B.

**Table 3. Associations between candidate drift indicators and post-deployment calibration degradation**

| **Drift indicator** | **System A βstd (95% CI)** | **System B βstd (95% CI)** | **System C βstd (95% CI)** | **System D βstd (95% CI)** |
| --- | --- | --- | --- | --- |
| **Population demographics shift** | -0.05 (-0.12 to 0.03) | -0.04 (-0.11 to 0.04) | -0.03 (-0.10 to 0.05) | -0.06 (-0.14 to 0.02) |
| **Outcome prevalence shift** | -0.18 (-0.28 to -0.08) | -0.16 (-0.26 to -0.06) | -0.07 (-0.16 to 0.02) | -0.08 (-0.18 to 0.01) |
| **Input missingness** | -0.42 (-0.56 to -0.29) | -0.38 (-0.52 to -0.25) | -0.24 (-0.37 to -0.11) | -0.21 (-0.34 to -0.08) |
| **Data latency** | -0.35 (-0.49 to -0.21) | -0.27 (-0.40 to -0.13) | -0.08 (-0.18 to 0.02) | -0.23 (-0.36 to -0.10) |
| **Feature availability change** | -0.22 (-0.35 to -0.09) | -0.34 (-0.48 to -0.20) | -0.19 (-0.31 to -0.07) | -0.37 (-0.51 to -0.23) |
| **Alert acknowledgement / override** | -0.07 (-0.16 to 0.02) | -0.06 (-0.15 to 0.03) | NA | NA |

βstd denotes the standardized regression coefficient from system-specific regression models with calibration slope as the dependent variable. Continuous drift indicators were standardized before model fitting. Negative coefficients indicate that higher values of the drift indicator were associated with lower calibration slope. Values are reported with 95% confidence intervals. NA indicates that the corresponding telemetry signal was not available or not applicable for that system.

Overall, workflow-associated indicators showed stronger and more consistent associations with post-deployment calibration degradation than population-related indicators.

**Methods Section 2.6 was revised with:**

For system-specific drift analyses, calibration slope was used as the primary dependent variable because calibration degradation was the earliest and most consistent post-deployment failure pattern. Associations between candidate drift indicators and calibration slope were estimated using standardized regression coefficients. Continuous drift indicators were standardized before model fitting. Coefficients are reported as standardized β estimates with 95% confidence intervals. Negative coefficients indicate an association between higher drift-indicator values and lower calibration slope.

**6. Results are verbose: The results section is split into many subsections with many qualitative figures/tables and claims made in the first half and more rigorous statistical analysis displayed in the second half. I recommend the author make the results section more compact/concise, introduce the statistical rigor up front, and avoid repetition. When reading the first half of the results, I agreed with the general findings, but I was puzzled why there were no rigorous statistics to justify the claims, only to find that some of this rigor is in the second half of the results.**

We reorganized the Results section to make the quantitative evidence appear earlier and to reduce repetition. The revised Results now present phase-level performance estimates and regression-based drift associations before the descriptive trajectory figures and failure-mode interpretation. Tables and figures were repositioned so that each appears immediately after the text in which it is first discussed.

**7. Figure 3: Missingness plot is interesting and seem to be a clear logical contributor to data drift and model failure. However, there is no explanation or interpretation of the data offered in regards to why missing data increases. Addition of an error analysis or summary of what pattern/kinds of missing data increases over time will improve the reader’s intuition of how this type of data drift contributes to model failure.**

We added a short missingness-pattern analysis to clarify which forms of missingness increased over time and how these patterns relate to calibration degradation. The revised Results now distinguish between intermittent input absence, delayed feature availability, and workflow-dependent documentation gaps, and links these patterns to the telemetry trajectories shown in Figure 3.

Missingness increases were not uniform across feature types. In Systems A and B, the largest increases occurred in time-sensitive EHR inputs, including recent vital-sign observations, laboratory values pending at inference time, and structured documentation fields dependent on clinical workflow completion. In Systems C and D, missingness increases were smaller but were concentrated in task-specific inputs, including delayed laboratory availability and incomplete imaging-derived feature extraction. These patterns indicate that increasing missingness reflected operational data availability and documentation timing rather than random feature loss. This supports the interpretation that missingness contributed to calibration degradation by altering the information available to the model at inference time.

**8. 3.5.1 Failure Mode 1: Author writes “During these periods, models preserved ranking ability but systematically misestimated absolute risk, constituting a clinically silent failure.” This is a good point to make regarding the trends, but it would be helpful to put this into a real clinical context. Specifically, consider gathering information from clinicians using this system on how much deviation from baseline performance for AUROC or calibration they would be willing to tolerate before they no longer trust the system. This may vary across different systems. Use this as a system-specific threshold and then compare time-to-event for how long it takes each of the measures to reach this threshold signifying major quality degradation and lack of trust/faith in the predictive modeling system. While the plots in Figure 1-5 provide strong evidence, they do not provide any indication of whether clinicians still use or trust the system or when the system becomes abandoned due to lack of accuracy/reliability.**

We expanded the clinical interpretation of Failure Mode 1 to clarify why calibration drift represents a clinically silent failure even when AUROC remains stable. We did not add a clinician-trust threshold or time-to-abandonment analysis because the retrospective dataset did not include clinician trust ratings, system-use abandonment events, or prospectively elicited acceptability thresholds. We now state this explicitly as a limitation and identify clinician-defined tolerance thresholds as an important target for prospective post-deployment evaluation.

**Section 3.5.1:**

Clinically, this failure mode is important because preserved ranking does not ensure correct absolute risk estimation. A model may continue to separate higher-risk from lower-risk patients while systematically overestimating or underestimating the probability of deterioration, sepsis, diagnosis, or adverse outcome. In threshold-based workflows, such miscalibration can change the number of patients crossing escalation thresholds, alter alert burden, or create false reassurance, even when AUROC remains within an apparently acceptable range.

**Section 4.4.3 Lack of prospectively elicited clinician tolerance thresholds:**

The study did not include prospectively elicited clinician tolerance thresholds for performance degradation or direct measures of clinician trust, reliance, or abandonment over time. Therefore, we did not estimate system-specific time-to-loss-of-trust thresholds. The clinical relevance of calibration drift was interpreted through its effect on absolute risk estimation and threshold-based decision-making rather than through measured clinician acceptability. Future prospective studies should define task-specific tolerance thresholds with clinical users and evaluate how calibration and discrimination changes affect trust, reliance, and continued system use.

**9. 3.5.2 Failure Mode 2: Author writes “Demographics and outcome prevalence remained stable…” but does not show any data to back up this claim. Please show figure or tables for demographic/outcome change over time or use a statistical model relating outcome variables (e.g. AUROC, calibration) with respect to time and adjust for confounders.**

We revised this section to avoid the unsupported claim that demographics and outcome prevalence “remained stable.” The revised manuscript now uses more cautious language and refers to workflow-associated degradation without comparable population-level change. We also added quantitative support in Table 3, which shows smaller and less consistent associations between population-related indicators and calibration degradation than between workflow-associated indicators and calibration degradation. We further clarified that this finding should not be interpreted as evidence that population change was absent or irrelevant.

A second failure mode was workflow-associated degradation without comparable population-level change. Population-related indicators showed smaller and less consistent associations with calibration degradation than workflow-associated indicators, whereas missingness, latency, and feature availability showed stronger associations across systems (Table 3).

This pattern indicates that operational changes in how data were generated, documented, or made available may destabilize deployed models even when broad population characteristics do not change to a comparable extent. The finding should be interpreted as evidence that workflow-associated telemetry was more closely aligned with

**10. 3.5 Characterization of post-deployment failure modes: Author writes “Several failure modes are not detectable through conventional validation or outcome-based monitoring despite clear safety implications.” Please elaborate on what these failure modes are and use your data to highlight several illustrative examples**

We revised Section 3.5 to clarify why the identified failure modes may be missed by conventional validation-era evidence or delayed under outcome-based monitoring. The revised text now links each failure mode to specific empirical examples from the study: early calibration drift despite preserved AUROC in Systems A and B, stronger associations between workflow telemetry and calibration degradation than population-related indicators, and delayed outcome-label availability across systems. These revisions clarify that the failure modes are derived from observed performance, telemetry, and label-availability patterns rather than from conceptual classification alone.

The quantitative and longitudinal analyses identified three recurrent post-deployment failure modes that would be incompletely captured by validation-era evidence alone and may be detected late under outcome-label-dependent monitoring: silent calibration failure under preserved discrimination, workflow-associated degradation without large population shift, and governance failure due to outcome-label latency. These failure modes were derived from the combined evidence in Tables 1–3 and Figures 1–5.

**3.5.1:**

In the present data, this pattern was most evident in Systems A and B, where early-deployment AUROC remained close to validation values, but calibration slopes and intercepts had already shifted away from validation-era estimates.

**3.5.3:**

Under outcome-based monitoring alone, degradation would therefore be detected only after a delay. This creates a governance gap in which systems may continue operating despite telemetry evidence of emerging performance degradation. In the present data, this was most relevant for Systems C and D, where outcome-label availability was delayed by weeks, while missingness, latency, and calibration trajectories were observable during deployment.

**11. Table 4: would be better if you showed the actual effect sizes with 95% CI for 3/6/9-month early phase, and then summarized the direction of the finding. Currently when we read the table, it is unclear how the thresholds were determined for Yes/No and the presentation of data feels too subjective.**

We revised Table 4 to replace categorical Yes/No summaries with quantitative sensitivity estimates. The revised table now reports validation-to-early-deployment differences in calibration slope and AUROC under 3-, 6-, and 9-month early deployment definitions, with 95% confidence intervals. We also revised the Methods to specify how these values were calculated: early deployment minus validation for each system, summarized as mean differences across systems with 95% confidence intervals.

**Methods:**

For deployment-phase sensitivity analyses, performance differences were calculated as the early deployment estimate minus the validation-phase estimate for each system and phase definition. System-specific differences were then summarized as mean differences across systems with 95% confidence intervals. Negative values indicate lower post-deployment performance compared with validation.

**3.6.1:**

The primary phase-level analysis defined early deployment as the first 6 months after clinical deployment and mature deployment as the period thereafter. To evaluate whether the observed validation–deployment gap depended on this boundary, phase-level comparisons were repeated using shorter and longer early deployment definitions of 3 months and 9 months.

Across phase definitions, calibration changed more consistently than discrimination. Validation-to-early-deployment differences in calibration slope were already evident using the 3-month definition and increased modestly under the 6- and 9-month definitions. In contrast, AUROC changes during early deployment remained smaller in magnitude, supporting the interpretation that calibration degradation emerged earlier than discrimination decline (Table 4).

**Table 4. Sensitivity analysis using alternative definitions of the early deployment phase**

|  | Role in analysis | Mean Δ calibration slope, validation to early deployment (95% CI) | Mean Δ AUROC, validation to early deployment (95% CI) | Direction of finding |
| --- | --- | --- | --- | --- |
| First 3 months after deployment | Sensitivity analysis | -0.08 (-0.11 to -0.05) | -0.01 (-0.02 to 0.00) | Calibration changed earlier than discrimination |
| First 6 months after deployment | Primary analysis | -0.11 (-0.14 to -0.08) | -0.02 (-0.03 to -0.01) | Calibration changed earlier than discrimination |
| First 9 months after deployment | Sensitivity analysis | -0.14 (-0.18 to -0.10) | -0.03 (-0.05 to -0.01) | Calibration changed earlier than discrimination |

The direction of the findings was unchanged across all early-deployment definitions. Calibration slope declined under each definition, with confidence intervals excluding zero, whereas AUROC differences were smaller and closer to zero. These results indicate that the main finding—earlier and more consistent calibration degradation relative to discrimination decline—was not an artifact of the selected 6-month primary phase definition.

**12. Table 7: Similar to my prior comments, the category values for Direction of association and Stability across specification need definition and explanation.**

We revised Table 6 to replace qualitative robustness categories with quantitative estimates. The revised table reports the range of standardized regression coefficients across alternative model specifications, the proportion of specifications preserving the expected direction of association, and the median change in explained variance. We also revised the Methods to specify how robustness across alternative regression specifications was summarized.

**Methods:**

Robustness analyses included leave-one-system-out models and alternative covariate specifications. For alternative regression specifications, robustness was summarized using the range of standardized regression coefficients across specifications, the proportion of specifications preserving the expected direction of association, and the median incremental explained variance associated with each signal category. Sensitivity analyses included alternative deployment-phase definitions, alternative temporal aggregation windows, and alternative regression specifications.

**Table 6:**

Associations between operational telemetry and calibration degradation were re-estimated using alternative regression specifications, including alternative covariate sets and exclusion of potentially collinear predictors (Table 6).

**Table 6. Robustness of telemetry–performance associations under alternative model specifications**

| **Signal category** | **βstd range across specifications** | **Specifications preserving expected direction** | **Median ΔR²** | **Robustness interpretation** |
| --- | --- | --- | --- | --- |
| Input missingness | -0.46 to -0.31 | 12/12 | 0.18 | Consistent negative association |
| Data latency | -0.39 to -0.24 | 12/12 | 0.13 | Consistent negative association |
| Feature availability change | -0.35 to -0.18 | 10/12 | 0.09 | Moderately consistent negative association |
| Population demographics | -0.09 to 0.04 | 6/12 | 0.03 | Inconsistent association |

Input missingness and data latency preserved negative associations with calibration slope in all tested specifications. Feature availability change preserved the expected direction in most specifications, whereas population demographics showed a small coefficient range, lower incremental explained variance, and inconsistent direction. These findings indicate that the telemetry–performance relationship was not driven by a single regression specification.

**13. Consider combining Sections 4 (Discussion) & 5 (Operational governance implications for deployed clinical AI). Much of the discussion currently re-iterates findings from results and confirmation of hypothesis, but does not pose any solution/suggestion–this is organized in a separate non-standard section. A more typical organization is to highlight key findings/results in the results, then discuss the implication of these results as well as potential insights into solutions or future work in the discussion. Organizationally, a point-by-point topical discussion of both implications + governance suggestions + future work is easier for readers to follow.**

We revised the manuscript structure to reduce repetition between the Discussion and the operational governance section. The separate “Operational governance implications” section was removed as a standalone section, and its key practical points were integrated into the Discussion. This keeps the governance implications in one location, avoids introducing a second discussion-style section after the limitations, and makes the manuscript more concise.

Monitoring outputs should be actionable, meaning that they are linked to predefined responses such as investigation, recalibration, workflow adjustment, threshold review, or temporary suspension. Without such linkages, even sophisticated monitoring risks becoming descriptive rather than protective. Deployment decisions should therefore be accompanied by explicit expectations about how performance may change over time, predefined monitoring plans linked to validated metrics, and criteria for reassessment or intervention when deviations occur. By identifying which signals are both observable and associated with degradation, this study provides a foundation for making post-deployment governance operational rather than aspirational.

**14. Conclusion: The conclusion section is too long and I recommend concluding with a single paragraph with top-level key points/findings/suggestions rather than a re-iteration of the discussion + governance sections. Your detailed narrative about conclusions is a better fit in the discussion section.**

We shortened the Conclusion to a single compact paragraph focused on the main findings and their practical implication. Discussion-like elaboration and governance detail were removed from the Conclusion and retained in the Discussion, where they are addressed more fully.

This longitudinal analysis of four deployed clinical AI systems shows that acceptable validation performance did not persist as a stable operational property after deployment. Across systems, degradation was progressive, often appeared first as calibration drift, and was more consistently associated with workflow-related telemetry signals such as missingness, latency, and feature availability than with broad population-level indicators. These findings support treating validation as a baseline operating reference rather than a durable safety guarantee. Post-deployment governance should therefore include continuous, calibration-aware monitoring and operational telemetry review, particularly when outcome labels are delayed or incomplete.

15. Data availability: If data availability is not possible due to sensitive clinical and operational information, would it be possible to de-identify the dataset? PLOS Data Policy (https://journals.plos.org/digitalhealth/s/data-availability) indicates that data should be shared whenever possible for maximal reproducibility of research. Also, please describe code availability for your statistical analysis so interested readers can understand your statistical analysis in greater depth.

The statistical analysis code used to generate the reported aggregate results, tables, and figures will be made available in a public repository upon publication. The repository will include scripts for temporal aggregation, performance-metric estimation, bootstrap confidence-interval calculation, regression modelling, sensitivity analyses, robustness analyses, and figure generation. Patient-level clinical and operational data will not be shared publicly because they contain sensitive information and are subject to privacy, security, and institutional governance restrictions. De-identified aggregate summary tables sufficient to reproduce the reported analyses may be made available from the corresponding author upon reasonable request and subject to institutional approvals.

**Reviewer #2: The manuscript takes up a timely and worthwhile question in clinical AI: why systems that appear to perform well before deployment often behave differently once they are introduced into real clinical settings. That problem matters, both methodologically and practically, and the paper makes a persuasive case that calibration drift and operational telemetry deserve closer attention in ongoing model surveillance. In broad terms, the manuscript is coherent, well organized, and situated in a relevant body of prior work.**

1. **The most immediate concern is methodological opacity. The manuscript refers to mixed-effects models, regression-based analyses, and lagged evaluations, but the statistical framework is described at too high a level to allow careful scrutiny or reproduction. At present, the reader is left to infer too much. The paper would benefit from a fuller account of the model structure, the covariates included, the unit and timing of temporal aggregation, the exact meaning of the lagged analyses, and the procedure used to separate workflow-related effects from population-related changes in performance. Without that level of detail, it is difficult to evaluate the strength of the empirical findings.**

We revised the Statistical analysis section to make the modelling framework more explicit. The revised Methods now define the system-time interval as the regression unit, specify the dependent variables, list the covariate blocks, describe the mixed-effects model structure, define the lagged analyses, and explain how population-related and workflow-associated indicators were compared using separate and combined covariate blocks, standardized coefficients, and changes in explained variance. This clarification was added to improve reproducibility and to make clear that workflow-related and population-related effects were compared as associated covariate blocks rather than interpreted as causal effects.

**Section 2.6:**

Regression analyses were performed at the system-time-interval level, where each observation represented one AI system within one post-deployment temporal interval. The primary dependent variable for drift-association analyses was calibration slope, because calibration degradation was the earliest and most consistent post-deployment failure pattern. Secondary dependent variables were calibration intercept, AUROC, and AUPRC where applicable.

Population-related indicators included demographic composition, available case-mix variables, and outcome prevalence. Workflow-associated indicators included input missingness, data latency, feature availability, inference volume, and available interaction measures. Continuous covariates were standardized before regression modelling.

The primary pooled model used a mixed-effects linear regression with a system-level random intercept. The general model form was:

*Performance*s,t = β0 + β1*time*s,t + β2*population indicators*s,t + β3*workflow indicators*s,t + us + εs,t,

where *s* denotes the AI system, *t* denotes the post-deployment time interval, *u*s is the system-level random intercept, and εs,t is the residual error.

To compare population-related and workflow-associated indicators, three model blocks were estimated: a population-only block, a workflow-only block, and a combined population-plus-workflow block. The relative contribution of each block was summarized using standardized regression coefficients, 95% confidence intervals, changes in explained variance, and consistency of effect direction across systems and robustness specifications.

Lagged analyses evaluated whether operational telemetry indicators in preceding time intervals were associated with subsequent performance metrics. For each system, one-interval and two-interval lagged versions of missingness, latency, and feature availability were created. These lagged telemetry variables were then related to subsequent calibration slope, calibration intercept, AUROC, and AUPRC using the same system-time-interval structure and mixed-effects framework as the primary regression analyses. Lagged analyses were used to assess temporal ordering between telemetry change and later performance degradation.

System-specific models were used to generate the standardized coefficients reported in Table 3, while pooled mixed-effects models and alternative model specifications were used for the robustness analyses summarized in Tables 5 and 6.

2. **A second issue involves the anonymization of the deployed systems. Some degree of abstraction is understandable, and perhaps necessary, but the current descriptions of Systems A through D are so general that interpretability suffers. As written, it is hard to judge how transferable the findings may be across settings or even how clinically distinct these systems really are. More structured information would help here. For example, the reader should ideally know something about the prediction horizon, the prevalence of the target outcome, the broad feature classes used by the models, and the way each system interacts with clinical workflow at deployment. Even if institution-specific identifiers cannot be disclosed, that kind of framing would make the results easier to interpret.**

We revised the system description to improve interpretability while preserving anonymization. Table 1 now includes additional structured information for each deployed system, including prediction horizon, target outcome prevalence, broad feature classes, and workflow integration mode. We also added a short explanatory sentence clarifying that these descriptors were included to support interpretation and transferability without disclosing vendor names, local identifiers, or institution-specific implementation details.

**Table 1. Clinical AI systems included in the empirical evaluation**

| **ID** | **Clinical task** | **Data modality** | **Deployment setting** | **Prediction horizon** | **Inference events, thousands** | **Outcome prevalence** | **Primary outcome** | **Typical outcome-label lag** | **Broad feature classes** | **Workflow integration mode** |
| --- | --- | --- | --- | --- | --- | --- | --- | --- | --- | --- |
| A | Early clinical deterioration risk prediction | EHR | Inpatient acute care | 24 hours | 1,240 | 8.7-9.1% | ICU transfer within 24 hours | 24-72 hours | Vital signs, recent laboratory values, medication and order activity, encounter context, nursing documentation | Passive risk score and threshold-based escalation list reviewed by ward team |
| B | Sepsis risk stratification | EHR | Emergency department and inpatient wards | 6–12 hours | 410 | 6.4-7.0% | Sepsis onset based on clinical diagnosis | 3-14 days | Vital signs, laboratory values, infection-related orders, medication administration, clinical documentation, prior utilization | Real-time alert integrated into emergency and ward workflow with acknowledgement / override logging |
| C | Diagnostic decision support | Laboratory data and structured EHR | Specialty outpatient clinics | Diagnostic episode / index visit | 96 | 14.2-14.8% | Confirmed diagnosis | 2-8 weeks | Laboratory panels, structured diagnoses, demographics, referral context, prior test history | Decision-support output available during diagnostic review; no interruptive alert |
| D | Outcome risk prediction | Imaging-derived features (CT/MR) | Radiology workflow | 30 days | 68 | 11.5-12.0% | Adverse outcome within 30 days | 7-30 days | Imaging-derived quantitative features, radiology protocol metadata, demographics, selected clinical context variables | Risk estimate generated after image processing and available in radiology reporting workflow |

In the primary analysis, early deployment refers to the first 6 months after deployment, and mature deployment refers to the subsequent observation period.

Additional system descriptors are reported to support interpretation of transferability across clinical contexts while preserving anonymization of local implementations, vendors, and institution-specific workflow details.

3. **I was also not fully convinced by the strength of some of the conclusions. The paper appears to suggest that workflow-related changes account for more of the observed degradation than population shift. That may be a reasonable interpretation of the patterns reported, but the present design is observational, and the language should reflect that limitation more carefully. What the analyses seem to show is association, possibly with temporal ordering, not causal demonstration. Tightening the wording on this point would make the argument more credible, not weaker.**

We revised the manuscript to avoid causal overstatement. The revised text now frames workflow-related telemetry as being more strongly and consistently associated with calibration degradation than population-related indicators, with some evidence of temporal ordering, rather than as a causal explanation. We also clarified in the Methods and Discussion that the observational design supports association and temporal alignment, not causal decomposition.

**Abstract:**

Workflow-associated changes in data availability and timing were more strongly and consistently associated with observed degradation than population-level indicators.

**Introduction, Hypothesis 2:**

Workflow-associated and operational changes in data generation, availability, and timing will show stronger associations with post-deployment performance divergence than population-level indicators alone, highlighting workflow-induced drift as a primary governance concern.

**Section 2.6 Statistical analysis:**

These analyses were interpreted as associations between covariate blocks and performance degradation, not as causal decomposition.

**Principal finding 3. Workflow-associated indicators were more strongly associated with degradation than population-related indicators**

Workflow-associated and operational telemetry signals showed stronger and more consistent associations with post-deployment calibration degradation than population-related indicators. Across systems, rising input missingness, data latency, and feature availability shifts aligned with – and often preceded – calibration degradation, whereas demographic and outcome-prevalence indicators showed smaller and less consistent associations. This supports a workflow-aware interpretation of post-deployment fragility: degradation may be linked to how data are produced within evolving workflows and information systems, not only to who the patients are [8,32,34,35].

**Section 4.3.3:**

A further implication concerns the importance of workflow-associated telemetry. Much existing guidance implicitly prioritizes monitoring for population drift — changes in demographics, case mix, or disease prevalence. While relevant, the results of this study indicate that workflow-aware signals were more consistently associated with degradation and may be more actionable for monitoring.

4. **The data availability statement also needs further attention. I understand the privacy constraints surrounding clinical and operational data, but the current statement does not seem fully aligned with PLOS expectations for reproducibility and data sharing. The authors should specify, as concretely as possible, what materials can be released for independent assessment. That might include code, derived summary tables, de-identified aggregate outputs, or a synthetic dataset designed to reproduce the analytical workflow. Right now, that part of the manuscript feels incomplete.**

We revised the Data availability statement to specify what reproducibility materials can be shared. The revised statement clarifies that patient-level clinical and operational data cannot be made publicly available because of privacy, security, and institutional governance restrictions. However, the statistical analysis code, derived aggregate summary tables, and a synthetic aggregate dataset reproducing the structure of the analyses will be made available to support independent assessment of the analytical workflow.

**Data availability statement**

Patient-level clinical and operational data cannot be shared publicly because they contain sensitive health information and operational metadata subject to privacy, security, and institutional governance restrictions. To support reproducibility, the authors will make available the statistical analysis code, derived aggregate summary tables used to generate the reported results, and an aggregate dataset that reproduces the structure of the analytical workflow without containing patient-level records or institution-identifiable information. De-identified aggregate outputs sufficient to reproduce the results will be made available online after the paper acceptance.

**Code availability statement**

Patient-level clinical and operational data cannot be shared publicly because they contain sensitive health information and operational metadata subject to privacy, security, and institutional governance restrictions. To support reproducibility, the authors will make available the statistical analysis code, derived aggregate summary tables used to generate the reported results, and an aggregate dataset that reproduces the structure of the analytical workflow without containing patient-level records or institution-identifiable information. De-identified aggregate outputs sufficient to reproduce the results will be made available online after the paper acceptance.

5. Relatedly, the paper should include a direct statement on ethical approval, institutional oversight, or governance arrangements for the use of clinical and operational data. Perhaps this information exists elsewhere in the submission package, but it should also be clear within the manuscript itself.

We expanded the ethics and governance statement to clarify institutional oversight for the use of clinical and operational data. The revised manuscript states that the retrospective study was approved by the local ethics committee, that informed consent was waived because the study used retrospective data and posed minimal risk, and that data access and analysis were conducted under institutional governance procedures using restricted access and de-identified or aggregate outputs.

**Ethics approval and consent to participate**

This retrospective study was reviewed and approved by the local Almazov Center Ethics Committee. The requirement for informed consent was waived because the study used retrospective data and posed minimal risk to participants. Access to clinical and operational data was governed by institutional data-use procedures. Analyses were conducted on restricted-access data, and reported outputs were limited to de-identified aggregate results. All data were handled in accordance with institutional policies and applicable privacy and data-protection regulations.

6. **There are also a few smaller issues. I noticed minor typographical problems, and some points are repeated across sections in ways that slightly dilute the overall impact. A careful line edit would likely resolve this without much difficulty.**

We performed a line edit to correct typographical issues, improve wording consistency, and reduce repetition across sections. In particular, we corrected duplicated punctuation, spacing errors, and overstrong or repetitive wording in the Abstract, Introduction, Discussion, and Conclusion.

**Reviewer #3:**

Through long-term longitudinal observation of four deployed clinical AI systems, this study effectively challenges the "Static Validation Paradigm" commonly adopted in the field. The "Post-Deployment Fragility" proposed by the authors is not an accidental failure at the model design level but rather a structural property that emerges when systems are embedded in dynamic clinical workflows. This insight is both academically profound and provides significant practical guidance. Particularly noteworthy is the core observation that "Calibration Drift" consistently precedes a decline in discrimination, providing an urgent and critical warning signal for clinical AI safety governance. However, the following two concerns are proposed for further exploration:

**1) The impact of the "Label Feedback Loop" on long-term stability and independent monitoring: When discussing "governance failure," how should we view the potential interference caused by the "Label Feedback Loop"? The manuscript notes that clinical data are not passively observed but are produced through sociotechnical processes where clinician interactions recursively influence both inputs and outcomes. When an AI system begins to guide clinical actions (e.g., an AI-predicted high risk leads to early intervention), this intervention changes the generation process of the outcome labels, causing performance audits to be "polluted" by Automation Bias. Given the existence of such "feedback pollution," can your advocated "Telemetry-first" monitoring signals (such as input missingness and data latency) maintain their independence as a safety baseline? Since these signals are observable at inference time and do not depend on outcome labels, can they still provide an "unbiased" measure of system health when the labels themselves have been compromised by the feedback loop?**

We expanded the Discussion to clarify how label feedback loops affect post-deployment monitoring. We now distinguish between outcome-dependent performance assessment, which can be biased when model outputs influence clinical actions and subsequent labels, and label-independent operational telemetry, which is observable at inference time before downstream intervention. We clarify that telemetry signals such as missingness, latency, and feature availability are not unbiased measures of clinical effectiveness, but they provide a less label-contaminated baseline for monitoring data-pipeline and workflow integrity. We also added this issue to the limitations, noting that the present study did not causally separate model-induced outcome changes from underlying clinical risk.

**Section 4.3.4:**

A related concern is label feedback. Once a deployed AI system influences clinical decisions, subsequent outcome labels may partly reflect model-guided intervention rather than the unmodified natural history of disease. For example, an early warning score that triggers earlier treatment may change the later outcome used to audit the model. This can bias outcome-dependent reassessment and make delayed labels difficult to interpret as independent ground truth. Telemetry-first monitoring does not solve this causal problem, but it reduces dependence on potentially contaminated outcome labels for early safety surveillance. Signals such as input missingness, data latency, feature availability, score-distribution change, and interaction logs are observable at or near inference time, before downstream clinical response is fully expressed. They should therefore be interpreted not as unbiased measures of clinical effectiveness, but as less label-dependent indicators of data-pipeline integrity, workflow stability, and changing operating conditions.

**Section 4.4.4:**

The analyses focus on detection and characterization of degradation rather than on organizational responses once degradation is identified. In real deployments, health systems may intervene through recalibration, workflow modification, retraining, or temporary suspension, potentially altering observed trajectories. In addition, model-generated predictions may influence clinician behavior and subsequent outcome labels, creating label feedback that complicates outcome-based reassessment. These adaptive responses and feedback mechanisms were not modeled explicitly.

**2) The universal evolution of "Post-Deployment Fragility" across different data modalities (e.g., Omics data): This study primarily focuses on EHR and imaging-derived features, yet a vast number of clinical models are now constructed using Omics data (e.g. PMID: 39710434, PMID: 39432561, PMID: 35136933). Omics and EHR data differ significantly in their collection, processing, and application. A point worth exploring is whether this "Post-Deployment Fragility" exists to an equal or even more pronounced degree in Omics-based models. In EHR scenarios, drift often stems from changes in clinical recording habits and data latency. In the field of Omics, while biological samples may remain relatively stable, the laboratory workflows (including sequencing platform updates, reagent batch effects, and bioinformatic pipeline iterations) essentially constitute the "sociotechnical processes" upon which the models rely. Would changes in these "technical telemetry signals" trigger a rapid failure in model calibration similar to what was observed in this study? Further discussion on the universality of this governance framework across different data modalities would greatly enhance the theoretical breadth of this research in the context of precision medicine governance.**

We expanded the Discussion to clarify how the concept of post-deployment fragility may extend to omics-based clinical AI and precision medicine models. We now note that, although omics inputs differ from EHR and imaging data, they are also generated through sociotechnical and technical workflows involving sample handling, sequencing or assay platforms, reagent lots, normalization methods, and bioinformatic pipeline versions. We added the reviewer-suggested omics references and clarified that, in omics-based deployment, telemetry-first monitoring would need to include technical telemetry such as platform version, batch effects, quality-control metrics, read depth or coverage, normalization parameters, and pipeline provenance. We present this as an implication for future work rather than as an empirical finding of the present study.

**Section 4.3.3:**

These findings support a telemetry-first monitoring approach in which operational indicators are used for early warning and triage, while outcome-based reassessment serves as confirmatory evidence once labels become available. Although the present study focused on EHR-, laboratory-, and imaging-derived systems, the same governance logic may extend to omics-based clinical AI models. Omics inputs are not passively observed biological facts; they are generated through laboratory and bioinformatic workflows involving sample handling, sequencing or assay platforms, reagent lots, read depth or coverage, normalization, batch correction, and pipeline versions. Recent omics-based diagnostic models illustrate both the promise of transferring single-cell or molecular signatures across bulk and single-cell sequencing data and the dependence of such models on platform compatibility, normalization, and cohort-specific processing choices

[45–47]. In this context, telemetry-first monitoring would need to include technical telemetry such as sample quality metrics, batch identifiers, assay or sequencing platform version, coverage or read-depth indicators, normalization parameters, and bioinformatic pipeline provenance. These signals may serve an analogous role to EHR missingness and latency by identifying changes in the data-generation process before outcome labels or clinical endpoints are available. The operational implication of this approach is summarized in Table 8, which translates the observed sequence of telemetry change, performance degradation, delayed labels, and governance response into a lifecycle monitoring framework.

**Section 4.4.2:**

For omics-based models, relevant telemetry may include assay platform, reagent batch, sample quality, sequencing depth, normalization procedures, batch-correction methods, and bioinformatic pipeline version; these were outside the empirical scope of the present analysis.
