# Supplementary material for: Validation is not enough: Longitudinal evidence of post-deployment fragility in clinical AI systems
Source: PLOS Digit Health. 2026 Jul 27;5(7):e0001534. doi: 10.1371/journal.pdig.0001534 (PMC13405297; doi:10.1371/journal.pdig.0001534)
Supplement: Attachment — Submitted filename: PLOS_response_reviewers_final.docx [file pdig.0001534.s002.docx]

**Response to Reviewers**

**Manuscript: Validation Is Not Enough: Longitudinal Evidence of Post-Deployment Fragility in Clinical AI Systems**

Revision 2

# Introductory response

Dear Editor and Reviewers,

We thank the reviewers for their careful evaluation of the revised manuscript and for the constructive suggestions that helped us improve the clarity, reproducibility, and organization of the paper. We have revised the manuscript to address the remaining structural concerns raised by Reviewer #4 and the reproducibility and interpretation issues raised by Reviewer #5.

In the revised manuscript, changes addressing Reviewer #4 are highlighted in green, and changes addressing Reviewer #5 are highlighted in blue. We also prepared the manuscript so that a clean version without highlighted changes can be submitted separately if required by the journal.

The main revisions are summarized below, followed by point-by-point responses to each reviewer comment.

| **Reviewer concern** | **Main revision made** |
| --- | --- |
| Reviewer #4: overlap between Methods, Results, and Discussion | Definitions, procedures, data sources, and summarization rules were moved or consolidated in the Methods; Results were revised to report empirical findings; governance interpretation was retained in the Discussion. |
| Reviewer #4: Section 3.1 / Table 1 deployment-characteristic assessment unclear | Section 2.2 now explains which deployment characteristics were extracted, why they were selected, from which data sources, and how they were summarized. |
| Reviewer #5: telemetry definitions insufficiently reproducible | Section 2.4 now defines input missingness, data latency, and feature availability and explains how routinely expected versus clinically conditional variables were interpreted. |
| Reviewer #5: calibration slope rationale and System B calibration | Section 2.7 now states that calibration slope was selected a priori; Section 2.6 clarifies that System B calibration used the continuous predicted risk score before alert thresholding. |
| Reviewer #5: inference events, patient counts, and bootstrap unit | Table 1 now includes distinct patient counts; Methods now specify patient-level clustered bootstrap resampling. |
| Reviewer #5: latency/missingness interpretation and primary drivers | Results now specify system-specific missingness/latency drivers and Table 4 summarizes the primary operational sources of telemetry change. |
| Reviewer #5: monitoring recommendation and duplication | Discussion now presents telemetry as the early-warning layer and calibration as confirmatory reassessment; duplicate calibration figure and redundant text were removed. |

# Reviewer #4

### Comment 4.1

The authors have made careful and detailed additions across the manuscript. However, these additions appear to have introduced some overlap between sections, and some of the structural concerns raised in earlier reviewer comments appear to remain. In particular, parts of the Results section include definitions, procedures, and classification criteria that may be more appropriately described in the Methods section. In addition, some interpretive statements that would fit better in the Discussion appear to be included in other sections. Clarifying the distinct roles of the Methods, Results, and Discussion sections would help reduce repetition and improve the flow and readability of the manuscript.

Regarding Section 3.1, "Empirical cohort and deployment characteristics," Section 2.2 describes the general overview of the target systems and their eligibility criteria. However, the Methods section does not appear to fully explain how the deployment characteristics presented in the Results were assessed. Specifically, it would be helpful to clarify which characteristics were extracted, why they were selected, from which data sources, and according to which definitions or summarization procedures.

**Response:**

We agree with the reviewer that the expanded revision required clearer separation between methodological specification, empirical reporting, and interpretation. We therefore revised the manuscript to sharpen the role of each section. Definitions, data sources, extraction procedures, and summarization rules are now specified in the Methods. The Results section has been revised to focus on observed system characteristics, longitudinal performance estimates, telemetry trajectories, system-specific telemetry drivers, and empirically derived failure patterns. Broader interpretation concerning governance, monitoring strategy, and lifecycle assurance is now reserved for the Discussion.

In response to the specific concern about Section 3.1, we expanded Section 2.2 to clarify how the deployment characteristics reported in Table 1 were assessed. The revised Methods now specify which characteristics were extracted, why these variables were selected, the data sources used, and how inference volume, distinct patient count, outcome prevalence, outcome-label lag, broad feature classes, and workflow integration mode were summarized. Section 3.1 was correspondingly kept descriptive, reporting empirical deployment characteristics rather than introducing the assessment procedure. We also revised Sections 3.4-3.6 to reduce interpretive and causal-sounding language and to keep governance-oriented interpretation in the Discussion.

**Changes made:**

**Manuscript change / excerpt:**

*Section 2.2 was expanded to state that deployment characteristics reported in Table 1 were extracted from model documentation, deployment records, inference logs, source-system metadata, feature specifications, and outcome-label definitions. The same section now lists the extracted characteristics and explains how inference volume, distinct patient count, outcome prevalence, typical outcome-label lag, broad feature classes, and workflow integration mode were summarized.*

**Manuscript change / excerpt:**

*Sections 3.1, 3.4, 3.5, and 3.6 were revised so that Results report observed deployment characteristics, longitudinal trajectories, telemetry drivers, and empirical failure patterns. Governance-oriented interpretation is retained in the Discussion, particularly Sections 4.2 and 4.3.*

# Reviewer #5

The authors present a study of clinically implemented AI systems to understand performance differences between validation and post-deployment epochs. Four separate systems with different use cases and data inputs were analyzed: these were intentionally and reasonably without sufficient detail to identify the systems or institution(s) being investigated. Post-deployment fragility is a very important topic in the field of predictive analytics and institutions implementing such systems for prospective clinical use. The findings of the manuscript are compelling in that they identify possible early indicators of decreased performance that may be evident before label-based performance tracking is available.

The authors have made substantial changes in response to prior reviews, and the manuscript is much improved as a result. The manuscript, though, still lacks sufficient transparency to be reproducible and would benefit from minor revisions below.

### Comment 5.1

The manuscript uses “input missingness”, “data latency”, and “feature availability” very many times throughout but never defines how these are quantified. The methods section should include sufficient detail so that a reader at a different institution could repeat this analysis. It is unclear, for example, how input missingness differs from feature availability, as both connote data not available. How did the authors quantify missingness across multiple different data modalities, some of which would not be expected to be missing (e.g., standard vital signs and labs) while others may be meaningfully missing (e.g., cultures or lactates drawn or medications administered only for suspicion of deterioration).

**Response:**

We agree that these telemetry variables required clearer operational definitions. We revised the Methods to define input missingness, data latency, and feature availability separately, because they capture related but distinct aspects of data availability at inference time. Input missingness is now defined at the individual feature-field level, feature availability at the broader feature-class level, and data latency as the time between source-system event time and availability to the AI system. We also clarified that not all absent data were interpreted in the same way: routinely expected measurements, such as scheduled vital signs or standard laboratory tests, were treated differently from clinically conditional features, such as cultures, lactate, or medications that may be absent because they were not clinically indicated. These revisions were added to make the telemetry analysis reproducible across institutions and modalities.

**Changes made:**

**Manuscript change / excerpt:**

*Section 2.4 now defines operational telemetry at inference time: input missingness as the proportion of expected model input fields unavailable within the prediction window; data latency as elapsed time from source-system timestamp to availability to the AI system; and feature availability as presence of at least one valid temporally eligible value within a broader feature class. The section also distinguishes routinely expected from clinically conditional variables.*

### Comment 5.2

On lines 327-329, the authors select calibration slope as the primary dependent variable “because calibration degradation was the earliest and most consistent post-deployment failure pattern.” It is not appropriate to select the primary analysis based on the results of the analysis. Is there no other reasoning for selecting calibration slope?

**Response:**

We agree that the previous wording could be interpreted as post hoc selection of the primary dependent variable. We revised the Methods to clarify that calibration slope was selected a priori because it reflects the reliability of absolute risk estimates used in threshold-based clinical decision support. This is directly relevant to escalation thresholds, alert burden, and safety monitoring, and cannot be assessed by discrimination metrics alone. The observation that calibration degradation emerged earlier and more consistently than discrimination decline is now presented only as a study finding, not as the rationale for selecting the primary endpoint.

**Changes made:**

**Manuscript change / excerpt:**

*Section 2.7 now states that the primary dependent variable for drift-association analyses was calibration slope, selected a priori because calibration reflects the reliability of absolute risk estimates used in threshold-based clinical decision support.*

### Comment 5.3

Please provide details of how calibration slope and intercept was determined for System B implemented as a real-time alert.

**Response:**

We clarified how calibration was estimated for System B despite its implementation as a real-time alert. The deployed workflow exposed a binary alert to clinicians, but the inference logs retained the underlying continuous predicted risk score before thresholding. Calibration slope and intercept were therefore estimated from the continuous predicted risk, not from the binary alert output. The alert threshold was kept fixed during the observation period and was used only to define the deployed alerting workflow, not to estimate calibration.

**Changes made:**

**Manuscript change / excerpt:**

*Section 2.6 now states that System B calibration analyses used the continuous predicted risk score recorded in the inference logs before application of the real-time alert threshold. Calibration slope and intercept were estimated by fitting a logistic calibration model using the observed binary outcome and logit-transformed predicted risk.*

### Comment 5.4

The Table 1 provides details of the clinical AI systems including inference events. It should also include the number of distinct patients.

**Response:**

We agree that inference-event counts alone do not describe the patient-level scale of each deployment, particularly because some patients may contribute multiple inference events. We revised Table 1 to include the number of distinct patients contributing at least one inference event for each system. This addition improves interpretation of deployment scale and helps distinguish repeated prediction frequency from the number of patients represented in the analysis.

**Changes made:**

**Manuscript change / excerpt:**

*Table 1 now includes a “Distinct patients, thousands” column. Section 2.2 also defines distinct patient count as the number of unique patients contributing at least one inference event.*

### Comment 5.5

Were bootstrap confidence intervals obtained by resampling by inference event or by patient?

**Response:**

We clarified the resampling unit used for bootstrap confidence intervals. Because patients could contribute more than one inference event, confidence intervals were estimated using patient-level clustered bootstrap resampling rather than independent resampling of inference events. In each bootstrap iteration, patients were sampled with replacement within each system and temporal interval, and all inference events belonging to sampled patients were retained. This approach preserves within-patient correlation across repeated predictions.

**Changes made:**

**Manuscript change / excerpt:**

*Section 2.6 now specifies that confidence intervals were estimated using patient-level clustered bootstrap resampling, with patients sampled with replacement within each system and temporal interval and all inference events belonging to sampled patients retained.*

### Comment 5.6

The data latency doubles across 18 months for every model (Figure 4). This should not happen for standard-of-care measurements (e.g., daily labs, periodic vital sign assessment) but may for diagnostic measurements taken for cause (e.g., lactic draw for suspicion of sepsis), and especially in response to an AI system. Is the “silent calibration” failure mode driven by feature selection that includes features not missing at random and, therefore, evident well before deployment even up to model development time? Or is it a change in clinical practice by using the AI system, as the authors suppose, in which case the AI system is working so long as the outcomes improve? Some of my confusion likely arises due to the lack of definition of data latency in the methods. Data are generated by care, but necessary care presumably is not being delayed. Proper interpretation cannot be made with the current information.

**Response:**

We agree that data latency required clearer definition and more cautious interpretation. We revised the Methods to define data latency as the interval between the source-system timestamp and availability of the corresponding input to the AI system at inference time. We also clarified that latency reflects model-input availability, not delayed clinical measurement, treatment, or care delivery. In the Results, we added a system-level interpretation of the observed latency trends. In the Limitations, we clarified that the study cannot determine whether latency changes resulted from background workflow evolution, clinically conditional measurement patterns, or clinician response to AI outputs. We therefore avoid interpreting increased latency as direct evidence of delayed care or as proof that the AI system caused the workflow change.

**Changes made:**

**Manuscript change / excerpt:**

*Sections 2.4 and 2.6 now define latency as model-input availability. Section 3.4 interprets latency sources by modality, and Section 4.4.4 states that operational telemetry was not interpreted as a direct measure of care quality and that the study could not distinguish background workflow evolution, clinically conditional measurement patterns, clinician response to AI outputs, or other organizational changes.*

### Comment 5.7

Similarly input missingness increases 50% (Figure 3). The interpretation of the finding of reduced calibration slope depends on whether that is missing vital signs, laboratory values, medications, documentation, demographics, etc.

**Response:**

We agree that the interpretation of increased input missingness depends on which input categories became unavailable. We revised the Results to specify the main feature groups contributing to increased missingness by system. The increase was not interpreted as uniform data loss. In EHR-based systems, missingness primarily involved recent vital signs, laboratory values pending at inference time, medication/order-derived variables, and structured documentation fields. In the diagnostic and imaging-derived systems, missingness was concentrated in delayed laboratory inputs and incomplete imaging-derived feature extraction. This clarification was added to support interpretation of the association between missingness and calibration slope.

**Changes made:**

**Manuscript change / excerpt:**

*Section 3.4 now describes the primary feature categories contributing to missingness by system, including vital signs, laboratory values, medication/order-derived variables, structured documentation, referral-context variables, prior-test information, imaging-derived quantitative features, and protocol metadata.*

### Comment 5.8

While it may be necessary to anonymize the systems and institutions, it would be informative to the reader for authors to identify the primary drivers of increased data latency and input missingness for each system.

**Response:**

We revised the Results to identify the primary system-specific drivers of increased input missingness and data latency while preserving institutional and vendor anonymity. A new summary table was added after the missingness and latency figures to distinguish EHR documentation, laboratory availability, medication/order-derived variables, imaging feature extraction, and protocol metadata as contributors to the observed telemetry trends. This addition clarifies that the observed changes were not uniform across systems or feature types.

**Changes made:**

**Manuscript change / excerpt:**

*A new Table 4 summarizes the primary drivers of increased input missingness and data latency by system and provides an interpretation of the likely operational source for each telemetry pattern.*

### Comment 5.9

The Discussion paragraph beginning at line 726 suggests calibration slopes should be used for post-deployment monitoring. I suggest removing this paragraph. In the following section the authors identify that it would be better, given the data presented in the manuscript, to track input missingness, data latency, etc. This especially since they don’t depend on outcome availability, and don’t rely on a biased calibration estimate from partial labeling or proxy events.

**Response:**

We agree with the reviewer. We revised this part of the Discussion to avoid implying that calibration slope should serve as the primary real-time monitoring signal. Calibration remains important for periodic outcome-based reassessment, but it depends on outcome availability and may be biased under partial labeling, proxy outcomes, or label feedback. We therefore reframed the governance recommendation: label-independent telemetry, including input missingness, data latency, feature availability, and score-distribution change, should provide the early-warning layer, while calibration slope and intercept should be used for confirmatory reassessment once sufficient outcome labels are available.

**Changes made:**

**Manuscript change / excerpt:**

*Section 4.3.2 now states that calibration slope and intercept should not be treated as primary real-time monitoring signals. Section 4.3.3 and Table 9 present label-independent telemetry as the early-warning layer and outcome-based calibration analysis as periodic confirmatory reassessment.*

### Comment 5.10

Figure 1 should start at 0.5 (random classification) and Figures 2 and 5 (duplicates) should start their y-axes at zero.

**Response:**

We agree. We revised the figure scaling and removed the duplicate calibration figure. Figure 1 now uses an AUROC y-axis beginning at 0.5, corresponding to random classification. The calibration plot now uses a y-axis beginning at 0. Because Figures 2 and 5 presented duplicate calibration-slope information, Figure 5 was removed and the remaining figure references were updated accordingly.

**Changes made:**

**Manuscript change / excerpt:**

*Figure 1 and Figure 2 were rescaled as requested. The duplicate Figure 5 was removed, and the remaining figure references were updated so that the Results refer to Figures 1-4 only.*

### Comment 5.11

There is duplication within the manuscript that distracts the reader and makes the manuscript harder to follow. Some examples follow. a. Section “Inclusion criteria for AI systems” on Page 11 duplicates eligibility criteria provided in the paragraph starting line 239. b. On lines 327-329, the authors select calibration slope as the primary dependent variable “because calibration degradation was the earliest and most consistent post-deployment failure pattern.” This statement is repeated verbatim on line 360. c. Figures 2 and 5 are duplicates.

**Response:**

We agree that duplication reduced readability. We revised the manuscript to remove repeated eligibility language, repeated calibration-slope justification, and duplicate calibration figures. The eligibility criteria are now introduced briefly and listed once in the Methods. The calibration-slope rationale is stated once as an a priori methodological choice, and redundant later wording was removed. We also removed the duplicate calibration figure and updated figure references so that each figure now presents distinct information.

**Changes made:**

**Manuscript change / excerpt:**

*Section 2.2 now presents eligibility criteria once; Section 2.7 contains a single a priori rationale for calibration slope; and the duplicate Figure 5 was removed.*

We believe these revisions improve the reproducibility, readability, and practical interpretability of the manuscript while preserving the necessary anonymization of systems and institutional workflow details. We thank the reviewers again for their constructive comments.

Sincerely,

Georgy Kopanitsa
