# Supplementary material for: Validation is not enough: Longitudinal evidence of post-deployment fragility in clinical AI systems
Source: PLOS Digit Health. 2026 Jul 27;5(7):e0001534. doi: 10.1371/journal.pdig.0001534 (PMC13405297; doi:10.1371/journal.pdig.0001534)
Supplement: Peer review history

# Peer review: Decision Letter 0

**DOI:** 10.1371/journal.pdig.0001534.r001

**Authors:** Louise A C Millard, Michael Winter

20 Apr 2026

PDIG-D-26-00413

Validation Is Not Enough: Longitudinal Evidence of Post-Deployment Fragility in Clinical AI Systems

PLOS Digital Health

Dear Dr. Kopanitsa,

Thank you for submitting your manuscript to PLOS Digital Health. After careful consideration, we feel that it has merit but does not fully meet PLOS Digital Health's publication criteria as it currently stands. Therefore, we invite you to submit a revised version of the manuscript that addresses the points raised during the review process.

Please submit your revised manuscript by Jun 19 2026 11:59PM. If you will need more time than this to complete your revisions, please reply to this message or contact the journal office at digitalhealth@plos.org. When you're ready to submit your revision, log on to https://www.editorialmanager.com/pdig/ and select the 'Submissions Needing Revision' folder to locate your manuscript file.

Please include the following items when submitting your revised manuscript:

* A letter that responds to each point raised by the editor and reviewer(s). You should upload this letter as a separate file labeled 'Response to Reviewers'. This file does not need to include responses to any formatting updates and technical items listed in the 'Journal Requirements' section below.

* A marked-up copy of your manuscript that highlights changes made to the original version. You should upload this as a separate file labeled 'Revised Manuscript with Track Changes'.

* An unmarked version of your revised paper without tracked changes. You should upload this as a separate file labeled 'Manuscript'.

If you would like to make changes to your financial disclosure, competing interests statement, or data availability statement, please make these updates within the submission form at the time of resubmission. Guidelines for resubmitting your figure files are available below the reviewer comments at the end of this letter.

We look forward to receiving your revised manuscript.

Kind regards,

Michael Winter

Academic Editor

PLOS Digital Health

Leo Anthony Celi

Editor-in-Chief

PLOS Digital Health

orcid.org/0000-0001-6712-6626

**Journal Requirements:**

1. Please provide an Author Summary. This should appear in your manuscript between the Abstract (if applicable) and the Introduction, and should be 150–200 words long. The aim should be to make your findings accessible to a wide audience that includes both scientists and non-scientists. Sample summaries can be found on our website under Submission Guidelines: [LINK]

https://journals.plos.org/digitalhealth/s/submission-guidelines#loc-parts-of-a-submission

2. In the online submission form, you indicated that "Data contain sensitive clinical and operational information and cannot be shared publicly due to privacy, security, and institutional governance restrictions. De-identified, aggregate summaries sufficient to reproduce the reported analyses may be made available from the corresponding author upon reasonable request and subject to institutional approvals.".

All PLOS journals now require all data underlying the findings described in their manuscript to be freely available to other researchers, either

1. In a public repository,

2. Within the manuscript itself, or

3. Uploaded as supplementary information.

This policy applies to all data except where public deposition would breach compliance with the protocol approved by your research ethics board. If your data cannot be made publicly available for ethical or legal reasons (e.g., public availability would compromise patient privacy), please explain your reasons by return email and your exemption request will be escalated to the editor for approval. Your exemption request will be handled independently and will not hold up the peer review process, but will need to be resolved should your manuscript be accepted for publication. One of the Editorial team will then be in touch if there are any issues.

If the reviewer comments include a recommendation to cite specific previously published works, please review and evaluate these publications to determine whether they are relevant and should be cited. There is no requirement to cite these works unless the editor has indicated otherwise.

**Additional Editor Comments (if provided):**

[Note: HTML markup is below. Please do not edit.]

**Reviewers' Comments:**

Reviewer's Responses to Questions

**Comments to the Author**

1. Does this manuscript meet PLOS Digital Health’s publication criteria? Is the manuscript technically sound, and do the data support the conclusions? The manuscript must describe methodologically and ethically rigorous research with conclusions that are appropriately drawn based on the data presented.

Reviewer #1: Yes

Reviewer #2: Yes

Reviewer #3: Yes

**********

2. Has the statistical analysis been performed appropriately and rigorously?

Reviewer #1: Yes

Reviewer #2: No

Reviewer #3: Yes

**********

3. Have the authors made all data underlying the findings in their manuscript fully available (please refer to the Data Availability Statement at the start of the manuscript PDF file)?

The PLOS Data policy requires authors to make all data underlying the findings described in their manuscript fully available without restriction, with rare exception. The data should be provided as part of the manuscript or its supporting information, or deposited to a public repository. For example, in addition to summary statistics, the data points behind means, medians and variance measures should be available. If there are restrictions on publicly sharing data—e.g. participant privacy or use of data from a third party—those must be specified.

Reviewer #1: No

Reviewer #2: No

Reviewer #3: Yes

**********

4. Is the manuscript presented in an intelligible fashion and written in standard English?

PLOS Digital Health does not copyedit accepted manuscripts, so the language in submitted articles must be clear, correct, and unambiguous. Any typographical or grammatical errors should be corrected at revision, so please note any specific errors here.

Reviewer #1: Yes

Reviewer #2: Yes

Reviewer #3: Yes

**********

5. Review Comments to the Author

Please use the space provided to explain your answers to the questions above. You may also include additional comments for the author, including concerns about dual publication, research ethics, or publication ethics. (Please upload your review as an attachment if it exceeds 20,000 characters)

Reviewer #1: Thank you for the opportunity to review this interesting study regarding post-deployment validation and drift in clinical AI/ML systems. The premise is well defined and the study approach is reasonable. The findings are particularly interesting and impactful, and I believe will help inform hospitals adopting AI/ML systems how to consider governance and accuracy issues once they have been deployed in clinical use.

However, I believe the manuscript should be improved prior to publication. Currently the manuscript is verbose and is organizationally difficult to read. Three main organizational issues that I felt impeded my comprehension: (1) results presented did not mirror described methods and method descriptions were not detailed enough, (2) discussion & governance implications are fractured into many subsections which makes it difficult to follow the progression of specific findings to governance suggestions, (3) Quantitative definitions for different temporal phases (e.g. “Early” vs. “Late”) and for categorical descriptors (e.g. “Weak” vs. “Strong”) are missing and lack rigor & justification for how data are placed into different categorical bins. I have attached my more detailed comments below.

Comments:

1. Methods: The methods tend to make general/generic statements and require more specificity to clearly describe the planned analyses reported in the results. For example, the results report sensitivity analysis on temporal aggregation, but this is not mentioned in the methods. For the reader, this creates a discordance between expected analyses and actual analyses that was reported.

2. Methods: Manuscript states “Analyses were conducted using standard statistical software with version-controlled scripts to ensure reproducibility.” Please provide more detail on the statistical software package used. Also, consider making your code publicly available along with the manuscript for transparency of the statistical analysis.

3. Results report different temporal periods or phases e.g. “Early Phase” or “Early Deployment”, etc. but it is unclear how these temporal phases are defined. Please standardize the definition for these and clearly define them and the principles by which they were chosen as well as justification to why your definitions are appropriate as opposed to some other arbitrary temporal phase definition.

4. Table 2: Please provide temporal range definitions for “Early Deployment” vs. “Mature Deployment”

5. Table 3: Please provide a caption or table legend that describes the definition of “Weak”, “Moderate”, “Strong”, and “Not Applicable”. Are these standardized effect sizes? Please describe in methods how these values are determined–there is currently a high-level but vague description. Please describe actual statistical methods, formulas, thresholds, definitions.

6. Results are verbose: The results section is split into many subsections with many qualitative figures/tables and claims made in the first half and more rigorous statistical analysis displayed in the second half. I recommend the author make the results section more compact/concise, introduce the statistical rigor up front, and avoid repetition. When reading the first half of the results, I agreed with the general findings, but I was puzzled why there were no rigorous statistics to justify the claims, only to find that some of this rigor is in the second half of the results.

7. Figure 3: Missingness plot is interesting and seem to be a clear logical contributor to data drift and model failure. However, there is no explanation or interpretation of the data offered in regards to why missing data increases. Addition of an error analysis or summary of what pattern/kinds of missing data increases over time will improve the reader’s intuition of how this type of data drift contributes to model failure.

8. 3.5.1 Failure Mode 1: Author writes “During these periods, models preserved ranking ability but systematically misestimated absolute risk, constituting a clinically silent failure.” This is a good point to make regarding the trends, but it would be helpful to put this into a real clinical context. Specifically, consider gathering information from clinicians using this system on how much deviation from baseline performance for AUROC or calibration they would be willing to tolerate before they no longer trust the system. This may vary across different systems. Use this as a system-specific threshold and then compare time-to-event for how long it takes each of the measures to reach this threshold signifying major quality degradation and lack of trust/faith in the predictive modeling system. While the plots in Figure 1-5 provide strong evidence, they do not provide any indication of whether clinicians still use or trust the system or when the system becomes abandoned due to lack of accuracy/reliability.

9. 3.5.2 Failure Mode 2: Author writes “Demographics and outcome prevalence remained stable…” but does not show any data to back up this claim. Please show figure or tables for demographic/outcome change over time or use a statistical model relating outcome variables (e.g. AUROC, calibration) with respect to time and adjust for confounders.

10. 3.5 Characterization of post-deployment failure modes: Author writes “Several failure modes are not detectable through conventional validation or outcome-based monitoring despite clear safety implications.” Please elaborate on what these failure modes are and use your data to highlight several illustrative examples

11. Table 4: would be better if you showed the actual effect sizes with 95% CI for 3/6/9-month early phase, and then summarized the direction of the finding. Currently when we read the table, it is unclear how the thresholds were determined for Yes/No and the presentation of data feels too subjective.

12. Table 7: Similar to my prior comments, the category values for Direction of association and Stability across specification need definition and explanation.

13. Consider combining Sections 4 (Discussion) & 5 (Operational governance implications for deployed clinical AI). Much of the discussion currently re-iterates findings from results and confirmation of hypothesis, but does not pose any solution/suggestion–this is organized in a separate non-standard section. A more typical organization is to highlight key findings/results in the results, then discuss the implication of these results as well as potential insights into solutions or future work in the discussion. Organizationally, a point-by-point topical discussion of both implications + governance suggestions + future work is easier for readers to follow.

14. Conclusion: The conclusion section is too long and I recommend concluding with a single paragraph with top-level key points/findings/suggestions rather than a re-iteration of the discussion + governance sections. Your detailed narrative about conclusions is a better fit in the discussion section.

15. Data availability: If data availability is not possible due to sensitive clinical and operational information, would it be possible to de-identify the dataset? PLOS Data Policy (https://journals.plos.org/digitalhealth/s/data-availability) indicates that data should be shared whenever possible for maximal reproducibility of research. Also, please describe code availability for your statistical analysis so interested readers can understand your statistical analysis in greater depth.

Reviewer #2: The manuscript takes up a timely and worthwhile question in clinical AI: why systems that appear to perform well before deployment often behave differently once they are introduced into real clinical settings. That problem matters, both methodologically and practically, and the paper makes a persuasive case that calibration drift and operational telemetry deserve closer attention in ongoing model surveillance. In broad terms, the manuscript is coherent, well organized, and situated in a relevant body of prior work.

That said, I would recommend major revision before publication is considered, because several parts of the paper remain underdeveloped and, in a few places, the claims seem to run ahead of what the study design can comfortably support.

The most immediate concern is methodological opacity. The manuscript refers to mixed-effects models, regression-based analyses, and lagged evaluations, but the statistical framework is described at too high a level to allow careful scrutiny or reproduction. At present, the reader is left to infer too much. The paper would benefit from a fuller account of the model structure, the covariates included, the unit and timing of temporal aggregation, the exact meaning of the lagged analyses, and the procedure used to separate workflow-related effects from population-related changes in performance. Without that level of detail, it is difficult to evaluate the strength of the empirical findings.

A second issue involves the anonymization of the deployed systems. Some degree of abstraction is understandable, and perhaps necessary, but the current descriptions of Systems A through D are so general that interpretability suffers. As written, it is hard to judge how transferable the findings may be across settings or even how clinically distinct these systems really are. More structured information would help here. For example, the reader should ideally know something about the prediction horizon, the prevalence of the target outcome, the broad feature classes used by the models, and the way each system interacts with clinical workflow at deployment. Even if institution-specific identifiers cannot be disclosed, that kind of framing would make the results easier to interpret.

I was also not fully convinced by the strength of some of the conclusions. The paper appears to suggest that workflow-related changes account for more of the observed degradation than population shift. That may be a reasonable interpretation of the patterns reported, but the present design is observational, and the language should reflect that limitation more carefully. What the analyses seem to show is association, possibly with temporal ordering, not causal demonstration. Tightening the wording on this point would make the argument more credible, not weaker.

The data availability statement also needs further attention. I understand the privacy constraints surrounding clinical and operational data, but the current statement does not seem fully aligned with PLOS expectations for reproducibility and data sharing. The authors should specify, as concretely as possible, what materials can be released for independent assessment. That might include code, derived summary tables, de-identified aggregate outputs, or a synthetic dataset designed to reproduce the analytical workflow. Right now, that part of the manuscript feels incomplete.

Relatedly, the paper should include a direct statement on ethical approval, institutional oversight, or governance arrangements for the use of clinical and operational data. Perhaps this information exists elsewhere in the submission package, but it should also be clear within the manuscript itself.

There are also a few smaller issues. I noticed minor typographical problems, and some points are repeated across sections in ways that slightly dilute the overall impact. A careful line edit would likely resolve this without much difficulty.

Overall, I think the manuscript has real value. The central question is important, and the paper has the makings of a meaningful contribution to the literature on post-deployment monitoring in clinical AI. Still, it needs more methodological detail, more careful framing of the claims, and a clearer approach to reporting and reproducibility before it is ready for publication.

Reviewer #3: Through long-term longitudinal observation of four deployed clinical AI systems, this study effectively challenges the "Static Validation Paradigm" commonly adopted in the field. The "Post-Deployment Fragility" proposed by the authors is not an accidental failure at the model design level but rather a structural property that emerges when systems are embedded in dynamic clinical workflows. This insight is both academically profound and provides significant practical guidance. Particularly noteworthy is the core observation that "Calibration Drift" consistently precedes a decline in discrimination, providing an urgent and critical warning signal for clinical AI safety governance. However, the following two concerns are proposed for further exploration:

1) The impact of the "Label Feedback Loop" on long-term stability and independent monitoring: When discussing "governance failure," how should we view the potential interference caused by the "Label Feedback Loop"? The manuscript notes that clinical data are not passively observed but are produced through sociotechnical processes where clinician interactions recursively influence both inputs and outcomes. When an AI system begins to guide clinical actions (e.g., an AI-predicted high risk leads to early intervention), this intervention changes the generation process of the outcome labels, causing performance audits to be "polluted" by Automation Bias. Given the existence of such "feedback pollution," can your advocated "Telemetry-first" monitoring signals (such as input missingness and data latency) maintain their independence as a safety baseline? Since these signals are observable at inference time and do not depend on outcome labels, can they still provide an "unbiased" measure of system health when the labels themselves have been compromised by the feedback loop?

2) The universal evolution of "Post-Deployment Fragility" across different data modalities (e.g., Omics data): This study primarily focuses on EHR and imaging-derived features, yet a vast number of clinical models are now constructed using Omics data (e.g. PMID: 39710434, PMID: 39432561, PMID: 35136933). Omics and EHR data differ significantly in their collection, processing, and application. A point worth exploring is whether this "Post-Deployment Fragility" exists to an equal or even more pronounced degree in Omics-based models. In EHR scenarios, drift often stems from changes in clinical recording habits and data latency. In the field of Omics, while biological samples may remain relatively stable, the laboratory workflows (including sequencing platform updates, reagent batch effects, and bioinformatic pipeline iterations) essentially constitute the "sociotechnical processes" upon which the models rely. Would changes in these "technical telemetry signals" trigger a rapid failure in model calibration similar to what was observed in this study? Further discussion on the universality of this governance framework across different data modalities would greatly enhance the theoretical breadth of this research in the context of precision medicine governance.

**********

6. PLOS authors have the option to publish the peer review history of their article (what does this mean?). If published, this will include your full peer review and any attached files.

**Do you want your identity to be public for this peer review?**  If you choose “no”, your identity will remain anonymous but your review may still be made public.

For information about this choice, including consent withdrawal, please see our Privacy Policy.

Reviewer #1: **Yes:**  Philip Chung

Reviewer #2: No

Reviewer #3: No

**********

[NOTE: If reviewer comments were submitted as an attachment file, they will be attached to this email and accessible via the submission site. Please log into your account, locate the manuscript record, and check for the action link "View Attachments". If this link does not appear, there are no attachment files.]

**Figure resubmission:**

While revising your submission, we strongly recommend that you use PLOS’s NAAS tool (https://ngplosjournals.pagemajik.ai/artanalysis) to test your figure files. NAAS can convert your figure files to the TIFF file type and meet basic requirements (such as print size, resolution), or provide you with a report on issues that do not meet our requirements and that NAAS cannot fix.

After uploading your figures to PLOS’s NAAS tool - https://ngplosjournals.pagemajik.ai/artanalysis, NAAS will process the files provided and display the results in the "Uploaded Files" section of the page as the processing is complete. If the uploaded figures meet our requirements (or NAAS is able to fix the files to meet our requirements), the figure will be marked as "fixed" above. If NAAS is unable to fix the files, a red "failed" label will appear above. When NAAS has confirmed that the figure files meet our requirements, please download the file via the download option, and include these NAAS processed figure files when submitting your revised manuscript.

**Reproducibility:**

To enhance the reproducibility of your results, we recommend that authors of applicable studies deposit laboratory protocols in protocols.io, where a protocol can be assigned its own identifier (DOI) such that it can be cited independently in the future. Additionally, PLOS ONE offers an option to publish peer-reviewed clinical study protocols. Read more information on sharing protocols at https://plos.org/protocols?utm_medium=editorial-email&utm_source=authorletters&utm_campaign=protocols

# Author comment: Author response to Decision Letter 1

**DOI:** 10.1371/journal.pdig.0001534.r002

4 May 2026

Attachment Submitted filename: response PLOS.docx

# Peer review: Decision Letter 1

**DOI:** 10.1371/journal.pdig.0001534.r003

**Authors:** Louise A C Millard, Michael Winter

8 Jun 2026

PDIG-D-26-00413R1

Validation Is Not Enough: Longitudinal Evidence of Post-Deployment Fragility in Clinical AI Systems

PLOS Digital Health

Dear Dr. Kopanitsa,

Thank you for submitting your manuscript to PLOS Digital Health. After careful consideration, we feel that it has merit but does not fully meet PLOS Digital Health's publication criteria as it currently stands. Therefore, we invite you to submit a revised version of the manuscript that addresses the points raised during the review process.

Please submit your revised manuscript by Jul 08 2026 11:59PM. If you will need more time than this to complete your revisions, please reply to this message or contact the journal office at digitalhealth@plos.org. When you're ready to submit your revision, log on to https://www.editorialmanager.com/pdig/ and select the 'Submissions Needing Revision' folder to locate your manuscript file.

Please include the following items when submitting your revised manuscript:

* A letter that responds to each point raised by the editor and reviewer(s). You should upload this letter as a separate file labeled 'Response to Reviewers'. This file does not need to include responses to any formatting updates and technical items listed in the 'Journal Requirements' section below.

* A marked-up copy of your manuscript that highlights changes made to the original version. You should upload this as a separate file labeled 'Revised Manuscript with Track Changes'.

* An unmarked version of your revised paper without tracked changes. You should upload this as a separate file labeled 'Manuscript'.

If you would like to make changes to your financial disclosure, competing interests statement, or data availability statement, please make these updates within the submission form at the time of resubmission. Guidelines for resubmitting your figure files are available below the reviewer comments at the end of this letter.

As the corresponding author, your ORCID iD is verified in the submission system and will appear in the published article. PLOS supports the use of ORCID, and we encourage all coauthors to register for an ORCID iD and use it as well. Please encourage your coauthors to verify their ORCID iD within the submission system before final acceptance, as unverified ORCID iDs will not appear in the published article. Only the individual author can complete the verification step; PLOS staff cannot verify ORCID iDs on behalf of authors.

We look forward to receiving your revised manuscript.

Kind regards,

Michael Winter

Academic Editor

PLOS Digital Health

Leo Anthony Celi

Editor-in-Chief

PLOS Digital Health

orcid.org/0000-0001-6712-6626

**Journal Requirements:**

If the reviewer comments include a recommendation to cite specific previously published works, please review and evaluate these publications to determine whether they are relevant and should be cited. There is no requirement to cite these works unless the editor has indicated otherwise.

**Additional Editor Comments (if provided):**

[Note: HTML markup is below. Please do not edit.]

**Reviewers' Comments:**

Reviewer's Responses to Questions

**Comments to the Author**

1. If the authors have adequately addressed your comments raised in a previous round of review and you feel that this manuscript is now acceptable for publication, you may indicate that here to bypass the “Comments to the Author” section, enter your conflict of interest statement in the “Confidential to Editor” section, and submit your "Accept" recommendation.

Reviewer #1: All comments have been addressed

Reviewer #4: (No Response)

Reviewer #5: (No Response)

**********

2. Does this manuscript meet PLOS Digital Health’s publication criteria? Is the manuscript technically sound, and do the data support the conclusions? The manuscript must describe methodologically and ethically rigorous research with conclusions that are appropriately drawn based on the data presented.

Reviewer #1: Yes

Reviewer #4: Yes

Reviewer #5: Yes

**********

3. Has the statistical analysis been performed appropriately and rigorously?

Reviewer #1: Yes

Reviewer #4: Yes

Reviewer #5: Yes

**********

4. Have the authors made all data underlying the findings in their manuscript fully available (please refer to the Data Availability Statement at the start of the manuscript PDF file)?

The PLOS Data policy requires authors to make all data underlying the findings described in their manuscript fully available without restriction, with rare exception. The data should be provided as part of the manuscript or its supporting information, or deposited to a public repository. For example, in addition to summary statistics, the data points behind means, medians and variance measures should be available. If there are restrictions on publicly sharing data—e.g. participant privacy or use of data from a third party—those must be specified.

Reviewer #1: Yes

Reviewer #4: Yes

Reviewer #5: Yes

**********

5. Is the manuscript presented in an intelligible fashion and written in standard English?

PLOS Digital Health does not copyedit accepted manuscripts, so the language in submitted articles must be clear, correct, and unambiguous. Any typographical or grammatical errors should be corrected at revision, so please note any specific errors here.

Reviewer #1: Yes

Reviewer #4: Yes

Reviewer #5: Yes

**********

6. Review Comments to the Author

Please use the space provided to explain your answers to the questions above. You may also include additional comments for the author, including concerns about dual publication, research ethics, or publication ethics. (Please upload your review as an attachment if it exceeds 20,000 characters)

Reviewer #1: My concerns have been fully addressed and I believe the manuscript to be substantially improved. This manuscript addresses a very impactful and clinically important problem in health care AI/ML and I believe the findings to be very insightful and guide practitioners in the field.

Reviewer #4: The authors have made careful and detailed additions across the manuscript. However, these additions appear to have introduced some overlap between sections, and some of the structural concerns raised in earlier reviewer comments appear to remain. In particular, parts of the Results section include definitions, procedures, and classification criteria that may be more appropriately described in the Methods section. In addition, some interpretive statements that would fit better in the Discussion appear to be included in other sections. Clarifying the distinct roles of the Methods, Results, and Discussion sections would help reduce repetition and improve the flow and readability of the manuscript.

Regarding Section 3.1, “Empirical cohort and deployment characteristics,” Section 2.2 describes the general overview of the target systems and their eligibility criteria. However, the Methods section does not appear to fully explain how the deployment characteristics presented in the Results were assessed. Specifically, it would be helpful to clarify which characteristics were extracted, why they were selected, from which data sources, and according to which definitions or summarization procedures.

Reviewer #5: The authors present a study of clinically implemented AI systems to understand performance differences between validation and post-deployment epochs. Four separate systems with different use cases and data inputs were analyzed: these were intentionally and reasonably without sufficient detail to identify the systems or institution(s) being investigated. Post-deployment fragility is a very important topic in the field of predictive analytics and institutions implementing such systems for prospective clinical use. The findings of the manuscript are compelling in that they identify possible early indicators of decreased performance that may be evident before label-based performance tracking is available.

The authors have made substantial changes in response to prior reviews, and the manuscript is much improved as a result. The manuscript, though, still lacks sufficient transparency to be reproducible and would benefit from minor revisions below.

1. The manuscript uses “input missingness”, “data latency”, and “feature availability” very many times throughout but never defines how these are quantified. The methods section should include sufficient detail so that a reader at a different institution could repeat this analysis. It is unclear, for example, how input missingness differs from feature availability, as both connote data not available. How did the authors quantify missingness across multiple different data modalities, some of which would not be expected to be missing (e.g., standard vital signs and labs) while others may be meaningfully missing (e.g., cultures or lactates drawn or medications administered only for suspicion of deterioration).

2. On lines 327-329, the authors select calibration slope as the primary dependent variable “because calibration degradation was the earliest and most consistent post-deployment failure pattern.” It is not appropriate to select the primary analysis based on the results of the analysis. Is there no other reasoning for selecting calibration slope?

3. Please provide details of how calibration slope and intercept was determined for System B implemented as a real-time alert.

4. The Table 1 provides details of the clinical AI systems including inference events. It should also include the number of distinct patients.

5. Were bootstrap confidence intervals obtained by resampling by inference event or by patient?

6. The data latency doubles across 18 months for every model (Figure 4). This should not happen for standard-of-care measurements (e.g., daily labs, periodic vital sign assessment) but may for diagnostic measurements taken for cause (e.g., lactic draw for suspicion of sepsis), and especially in response to an AI system. Is the “silent calibration” failure mode driven by feature selection that includes features not missing at random and, therefore, evident well before deployment even up to model development time? Or is it a change in clinical practice by using the AI system, as the authors suppose, in which case the AI system is working so long as the outcomes improve? Some of my confusion likely arises due to the lack of definition of data latency in the methods. Data are generated by care, but necessary care presumably is not being delayed. Proper interpretation cannot be made with the current information.

7. Similarly input missingness increases 50% (Figure 3). The interpretation of the finding of reduced calibration slope depends on whether that is missing vital signs, laboratory values, medications, documentation, demographics, etc.

8. While it may be necessary to anonymize the systems and institutions, it would be informative to the reader for authors to identify the primary drivers of increased data latency and input missingness for each system.

9. The Discussion paragraph beginning at line 726 suggests calibration slopes should be used for post-deployment monitoring. I suggest removing this paragraph. In the following section the authors identify that it would be better, given the data presented in the manuscript, to track input missingness, data latency, etc. This especially since they don’t depend on outcome availability, and don’t rely on a biased calibration estimate from partial labeling or proxy events.

10. Figure 1 should start at 0.5 (random classification) and Figures 2 and 5 (duplicates) should start their y-axes at zero.

11. There is duplication within the manuscript that distracts the reader and makes the manuscript harder to follow. Some examples follow.

a. Section “Inclusion criteria for AI systems” on Page 11 duplicates eligibility criteria provided in the paragraph starting line 239.

b. On lines 327-329, the authors select calibration slope as the primary dependent variable “because calibration degradation was the earliest and most consistent post-deployment failure pattern.” This statement is repeated verbatim on line 360.

c. Figures 2 and 5 are duplicates.

**********

7. PLOS authors have the option to publish the peer review history of their article (what does this mean?). If published, this will include your full peer review and any attached files.

**Do you want your identity to be public for this peer review?**  If you choose “no”, your identity will remain anonymous but your review may still be made public.

For information about this choice, including consent withdrawal, please see our Privacy Policy.

Reviewer #1: **Yes:**  Philip Chung

Reviewer #4: No

Reviewer #5: No

**********

[NOTE: If reviewer comments were submitted as an attachment file, they will be attached to this email and accessible via the submission site. Please log into your account, locate the manuscript record, and check for the action link "View Attachments". If this link does not appear, there are no attachment files.]

**Figure resubmission:**

While revising your submission, we strongly recommend that you use PLOS’s NAAS tool (https://ngplosjournals.pagemajik.ai/artanalysis) to test your figure files. NAAS can convert your figure files to the TIFF file type and meet basic requirements (such as print size, resolution), or provide you with a report on issues that do not meet our requirements and that NAAS cannot fix.

After uploading your figures to PLOS’s NAAS tool - https://ngplosjournals.pagemajik.ai/artanalysis, NAAS will process the files provided and display the results in the "Uploaded Files" section of the page as the processing is complete. If the uploaded figures meet our requirements (or NAAS is able to fix the files to meet our requirements), the figure will be marked as "fixed" above. If NAAS is unable to fix the files, a red "failed" label will appear above. When NAAS has confirmed that the figure files meet our requirements, please download the file via the download option, and include these NAAS processed figure files when submitting your revised manuscript.

**Reproducibility:**

To enhance the reproducibility of your results, we recommend that authors of applicable studies deposit laboratory protocols in protocols.io, where a protocol can be assigned its own identifier (DOI) such that it can be cited independently in the future. Additionally, PLOS ONE offers an option to publish peer-reviewed clinical study protocols. Read more information on sharing protocols at https://plos.org/protocols?utm_medium=editorial-email&utm_source=authorletters&utm_campaign=protocols

# Author comment: Author response to Decision Letter 2

**DOI:** 10.1371/journal.pdig.0001534.r004

11 Jun 2026

Attachment Submitted filename: PLOS_response_reviewers_final.docx

# Editor report: Decision Letter 2

**DOI:** 10.1371/journal.pdig.0001534.r005

**Authors:** Louise A C Millard, Michael Winter

15 Jun 2026

Validation Is Not Enough: Longitudinal Evidence of Post-Deployment Fragility in Clinical AI Systems

PDIG-D-26-00413R2

Dear Dr Kopanitsa,

We are pleased to inform you that your manuscript 'Validation Is Not Enough: Longitudinal Evidence of Post-Deployment Fragility in Clinical AI Systems' has been provisionally accepted for publication in PLOS Digital Health.

Before your manuscript can be formally accepted you will need to complete some formatting changes, which you will receive in a follow-up email from a member of our team.

Please note that your manuscript will not be scheduled for publication until you have made the required changes, so a swift response is appreciated.

IMPORTANT: The editorial review process is now complete. PLOS will only permit corrections to spelling, formatting or significant scientific errors from this point onwards. Requests for major changes, or any which affect the scientific understanding of your work, will cause delays to the publication date of your manuscript.

If your institution or institutions have a press office, please notify them about your upcoming paper to help maximize its impact. If they'll be preparing press materials, please inform our press team as soon as possible -- no later than 48 hours after receiving the formal acceptance. Your manuscript will remain under strict press embargo until 2 pm Eastern Time on the date of publication. For more information, please contact digitalhealth@plos.org.

Thank you again for supporting Open Access publishing; we are looking forward to publishing your work in PLOS Digital Health.

Best regards,

Michael Winter

Section Editor

PLOS Digital Health

***********************************************************

**Additional Editor Comments (if provided):**

**Reviewer Comments (if any, and for reference):**
